# Nrf2 plays a critical role in the metabolic response during and after spaceflight

**DOI:** 10.1038/s42003-021-02904-6

**Published:** 2021-12-09

**Authors:** Akira Uruno, Daisuke Saigusa, Takafumi Suzuki, Akane Yumoto, Tomohiro Nakamura, Naomi Matsukawa, Takahiro Yamazaki, Ristumi Saito, Keiko Taguchi, Mikiko Suzuki, Norio Suzuki, Akihito Otsuki, Fumiki Katsuoka, Eiji Hishinuma, Risa Okada, Seizo Koshiba, Yoshihisa Tomioka, Ritsuko Shimizu, Masaki Shirakawa, Thomas W. Kensler, Dai Shiba, Masayuki Yamamoto

**Affiliations:** 1 Department of Integrative Genomics, Tohoku Medical Megabank Organization, Tohoku University, Sendai, Japan; 2 Department of Medical Biochemistry, Tohoku University Graduate School of Medicine, Sendai, Japan; 3 JEM Utilization Center, Human Spaceflight Technology Directorate, JAXA, Tsukuba, Japan; 4 Department of Health Record Informatics, Tohoku Medical Megabank Organization, Tohoku University, Sendai, Japan; 5 Laboratory of Oncology, Pharmacy Practice and Sciences, Graduate School of Pharmaceutical Sciences, Tohoku University, Sendai, Japan; 6 Advanced Research Center for Innovations in Next-GEneration Medicine (INGEM), Tohoku University, Sendai, Japan; 7 Center for Radioisotope Sciences, Tohoku University Graduate School of Medicine, Sendai, Japan; 8 Division of Oxygen Biology, Tohoku University Graduate School of Medicine, Sendai, Japan; 9 Department of Molecular Hematology, Tohoku University Graduate School of Medicine, Sendai, Japan; 10 Translational Research Program, Fred Hutchinson Cancer Research Center, Seattle, WA USA

**Keywords:** Lipidomics, Metabolomics

## Abstract

Space travel induces stresses that contribute to health problems, as well as inducing the expression of Nrf2 (NF-E2-related factor-2) target genes that mediate adaptive responses to oxidative and other stress responses. The volume of epididymal white adipose tissue (eWAT) in mice increases during spaceflight, a change that is attenuated by *Nrf2* knockout. We conducted metabolome analyses of plasma from wild-type and *Nrf2* knockout mice collected at pre-flight, in-flight and post-flight time points, as well as tissues collected post-flight to clarify the metabolic responses during and after spaceflight and the contribution of Nrf2 to these responses. Plasma glycerophospholipid and sphingolipid levels were elevated during spaceflight, whereas triacylglycerol levels were lower after spaceflight. In wild-type mouse eWAT, triacylglycerol levels were increased, but phosphatidylcholine levels were decreased, and these changes were attenuated in *Nrf2* knockout mice. Transcriptome analyses revealed marked changes in the expression of lipid-related genes in the liver and eWAT after spaceflight and the effects of *Nrf2* knockout on these changes. Based on these results, we concluded that space stress provokes significant responses in lipid metabolism during and after spaceflight; Nrf2 plays critical roles in these responses.

## Abstract (web summary)

Akira Uruno et al. report the volume of epidydimal white adipose tissue and plasma glycerophospholipid and sphingolipid levels in mice increase during spaceflight. These metabolic and physiological changes were largely driven by Nrf2 (NF-E2-related factor-2), ultimately providing a mechanistic target for altering lipid metabolism on Earth and the Great Beyond.

## Introduction

Space stresses, including microgravity and cosmic radiation, are known to evoke various health problems in the body. Salient examples in astronauts are skeletal muscle loss, osteoporosis, central nervous system damage, optic nerve oedema, body weight changes and insulin resistance^1–4^. Molecular and cellular characterizations of these responses to space stress converge to the conclusion that these phenotypes are partially attributable to increased oxidative stress and DNA damage, as well as dysregulation of mitochondria and changes in telomeres, epigenetic factors, microbiomes and gene regulation^5^. While increased insights into some of the biological changes that occur during spaceflight have been reported, the mechanisms underlying the regulation of body weight and fat mass volume remain controversial. For instance, some astronauts display a loss of body weight and fat mass volume^1,3,6,7^, while others showed increased or unchanged body mass or comparable scores for related parameters during spaceflight^1,8–10^. Previous studies in mice revealed that lipids accumulated in the liver after spaceflight^11^. Thus, the available evidence suggests that body weight and lipid metabolism are subject to intricate regulation during and after spaceflight and that metabolic responses to space stresses are essential for the precise understanding of health problems that arise during and after spaceflight.

Nrf2 (NF-E2-related factor-2) is a member of the CNC family of transcription factors^12^ and regulates the expression of a battery of genes that contribute to the protection of cells against oxidative and xenobiotic stresses, including radiation^13–18^. Nrf2 also plays important roles in the regulation of metabolism-related genes, such as those involved in glycogen utilization, gluconeogenesis, the pentose phosphate pathway and lipid metabolism^19–21^. Urinary excretion of the oxidative stress marker 8-hydroxy-2′-deoxyguanosine in astronauts^1,22^ increases after habitation in the International Space Station (ISS)^23^, suggesting that Nrf2 activity may be upregulated in response to the elevated oxidative stress during spaceflight.

We conducted the Mouse Habitat Unit-3 (MHU-3) project that sent six *Nrf2* knockout (Nrf2 KO) mice and six wild-type (WT) mice into space to delineate the roles of Nrf2 during and after spaceflight^24,25^. These Nrf2 KO and WT mice were housed in the Japanese Experiment Module “Kibo” in the ISS for 31 days. In the course of initial analyses, we found that Nrf2 activity is indeed induced by space travel and that both the weight and lipid droplet size of epididymal white adipose tissue (eWAT) are increased in WT mice after spaceflight. In contrast, Nrf2 deletion represses eWAT weight gain and lipid droplet enlargement, indicating the contribution of Nrf2 to the metabolic responses in mice exposed to spaceflight.

Blood samples obtained from mice during their stay aboard the ISS are critically important to more precisely understand the dynamics of metabolic changes provoked by the stresses of space travel. We propose that blood collection at a minimum of three time points, i.e., pre-flight, in-flight and post-flight, is necessary for informative metabolite analyses. However, while a number of reports have described blood collections before and/or after spaceflight^26–29^, obtaining blood from mice during their time in space has been technically difficult, and the collection of these samples has not been reported previously. For the MHU-3 project, we developed a special method that enables blood collection from the mouse tail during spaceflight^24^. Therefore, we were able to pursue elaborate metabolome analyses involving blood samples collected at three time points in the MHU-3 project. Two technological advances further supported the MHU-3 project. One is that a system for monitoring mouse food consumption and water drinking coupled with single cage housing technology has been developed to precisely analyse mouse metabolism during spaceflight^24,30^. The other is that a video downlink system has been developed for veterinarians to monitor real-time mouse health conditions^31^. Although we reported the preliminary results of the plasma metabolome analysis of five metabolites in our initial report^24^, we wished to conduct a more comprehensive study of the metabolic responses during spaceflight.

Therefore, in this study, we performed ultrahigh-performance liquid chromatography triple quadrupole MS (UHPLC-MS/MS) for a comprehensive metabolomic analyses of plasma collected from the tail to evaluate metabolic responses during and after spaceflight. We also conducted metabolome analyses of post-flight tissue samples, specifically, eWAT lipidomics and brain imaging, using UHPLC Fourier transform MS (UHPLC-FTMS) and matrix-assisted laser desorption/ionization MS imaging (MALDI-MSI) systems, respectively. Of the many salient observations, we found that changes in lipid metabolites in the plasma and tissues were particularly significant. For instance, in WT mice, the plasma levels of glycerophospholipids, sphingolipids and cholesteryl esters (CEs) were markedly increased in the in-flight samples, whereas those of triacylglycerols (TGs) were decreased in post-flight samples. These metabolic responses during spaceflight were substantially abrogated in the plasma of Nrf2 KO mice. We also performed RNA sequencing (RNA-seq) analyses and documented altered expression of metabolism-related genes in the liver and eWAT after spaceflight, as well as the underlying contributions of Nrf2 to these responses. This study supports our hypothesis that space stresses induce specific and severe metabolic responses and that Nrf2 plays important roles in the adaptive response to stress during and after spaceflight.

## Results

### Plasma collection in the MHU-3 project

**Fig. 1:**
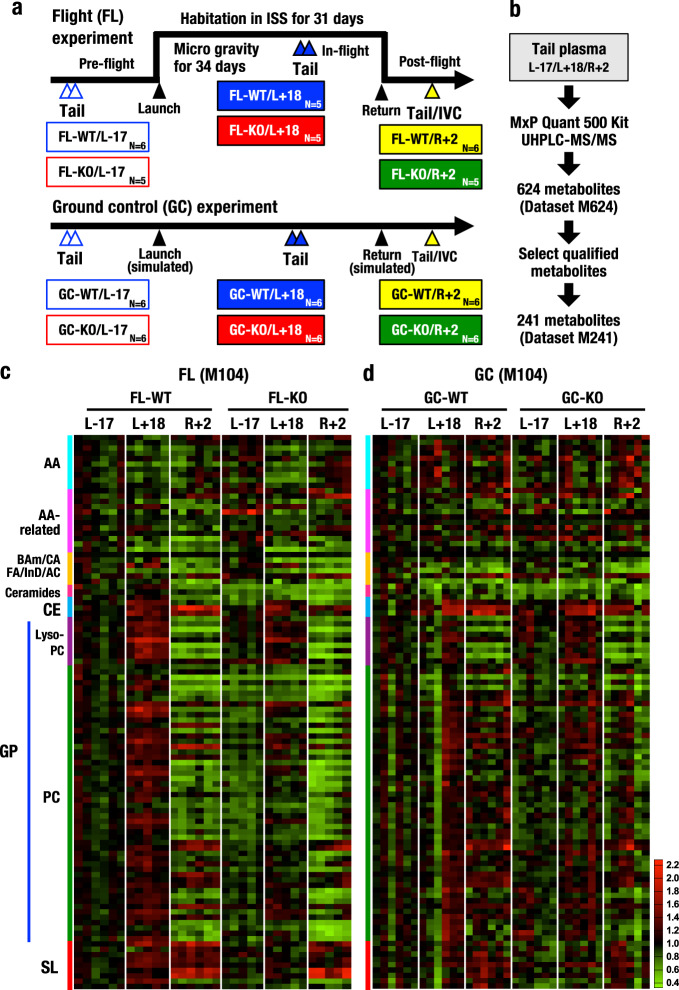
Plasma collection and metabolome analyses in the MHU-3 project. **a** Schematic presentation of the MHU-3 project in which spaceflight (FL) and ground control (GC) experiments were performed. Blood samples were collected from the mouse tail at pre-flight (L-17; 17 days before launch), in-flight (L+18; 18 days after launch) and post-flight (R+2; 2 days after return) time points. The numbers of mice analysed in this study are noted. **b** Flow chart of metabolome analyses of tail plasma samples collected at three time points. Plasma samples were submitted for the measurement of 624 metabolites (dataset M624) using UHPLC-MS/MS and an MxP Quant 500 kit. From the M624 dataset, 241 metabolites were qualified and selected for further review (M241). **c**, **d** Heatmap analyses for 104 metabolites selected from M241 for FL (**c**) and GC (**d**) experiments with a coefficient of variation (CV) value ≤30% (dataset M104). Means of FL-WT/L-17 (**c**) or GC-WT/L-17 (**d**) were set to one. The results of M104 are aligned in columns by ID, as described in Supplementary Data 2. AA amino acids, AA-related amino acid-related metabolites, BAm biogenic amines, CA carboxylic acids, FA fatty acids, InD indole derivatives, AC acylcarnitines, CE cholesteryl esters, LysoPC lysophosphatidylcholines, PC phosphatidylcholines, SL sphingolipids.

Six WT and six Nrf2 KO mice were launched into space and stayed aboard the ISS for 31 days^24^ (Fig. 1a). Blood samples were collected from the tail at three time points, i.e., pre-flight (17 days before launch; referred to as L-17), in-flight (18 days after launch; L+18), and post-flight (2 days after return; R+2). Blood samples were also collected from the inferior vena cava (IVC) at R+2 under anaesthesia. We conducted a ground control (GC) experiment in a similar manner to the spaceflight (FL) experiment. Blood collection from FL-WT mouse No. 8 (FL08) at L+18 was omitted upon recommendation of the veterinarians due to minor injury of the tail. In addition, FL-KO mouse No. 4 (FL04) suffered intestinal haemorrhage during the return flight^24^. We analysed the levels of ketone bodies in IVC blood samples from individual mice at R + 2 using NMR to determine whether the data from mouse FL04 needed to be excluded. Mouse FL04 displayed extremely elevated levels of ketone bodies compared with the other 23 mice (Supplementary Fig. 1a-c), indicating that the metabolic conditions of mouse FL04 were different from those of the other mice. Thus, the data from mouse FL04 were excluded from this study. Collectively, five samples were collected at L+18 and six samples were collected at L-17 and R+2 from the FL-WT group; five samples were collected from the FL-KO group at each of the three time points.

### Plasma lipid levels are elevated during spaceflight depending on the Nrf2 genotype

We performed plasma metabolome analyses using tail blood samples to evaluate metabolic responses during and after spaceflight. As we were only able to collect small volumes of plasma from the tails, we applied a UHPLC-MS-MS method and measured the plasma levels of 624 metabolites (dataset M624, Fig. 1b). We classified metabolites into four groups according to the criteria established by MetIDQ Oxygen software provided by the manufacturer (Biocrates Life Sciences), which mainly assessed the reliability of the data. Group 1 included metabolites below the limit of detection, Group 2 included metabolites above the limit of internal standards, Group 3 included metabolites below the lower limit of quantification or above the upper limit of quantification, and Group 4 included metabolites within the lower and upper limits of quantification. We then selected 241 qualified metabolites in Groups 3 and 4 (dataset M241) for further analysis.

Figure 1c presents a heatmap showing an overview of metabolic responses identified in the MHU-3 project. We refined the M241 dataset further and selected 104 metabolites based on the criterion that the coefficient of variation (CV) was 30% or less (dataset M104). The mean value of the FL-WT group at L-17 was set to 1.0 for the FL group (Fig. 1c) and that of the GC-WT group at L-17 was set to 1.0 for the GC group (Fig. 1d). In FL-WT mice, the plasma levels of lipids, especially cholesteryl esters, glycerophospholipids [including lysophosphatidylcholines (LysoPCs) and phosphatidylcholines (PCs)] and sphingolipids [including sphingomyelins (SMs)], were markedly increased in the L+18 in-flight samples compared to the L-17 pre-flight samples. Notably, the levels of many glycerophospholipids in R+2 post-flight samples decreased below the levels in L-17 samples in FL-WT mice, but the levels of sphingolipids and cholesteryl esters were further increased at R+2 compared to L-17 and L+18.

An intriguing observation is that the increased levels of cholesteryl esters, glycerophospholipids and sphingolipids in L+18 FL-WT mice were markedly attenuated in FL-KO mice. These results indicate that plasma levels of cholesteryl esters, glycerophospholipids and sphingolipids were elevated during spaceflight and that Nrf2 contributed to the increased levels of these lipids in the L+18 samples. However, the decreases in the levels of these lipids in R+2 post-flight samples appeared to be independent of Nrf2 activity. We surmise that the return to Earth gravity must impose severe stress for the mice and provoke these changes.

Figure 1d presents the heatmap showing an overview of the metabolic response observed in the GC experiment. We found that M104 metabolite levels were not significantly altered during the simulated flight. These data further support our conclusion that the increases in the plasma levels of cholesteryl esters, glycerophospholipids and sphingolipids during spaceflight are dependent on Nrf2.

### Plasma triacylglycerol levels decrease during spaceflight

**Fig. 2:**
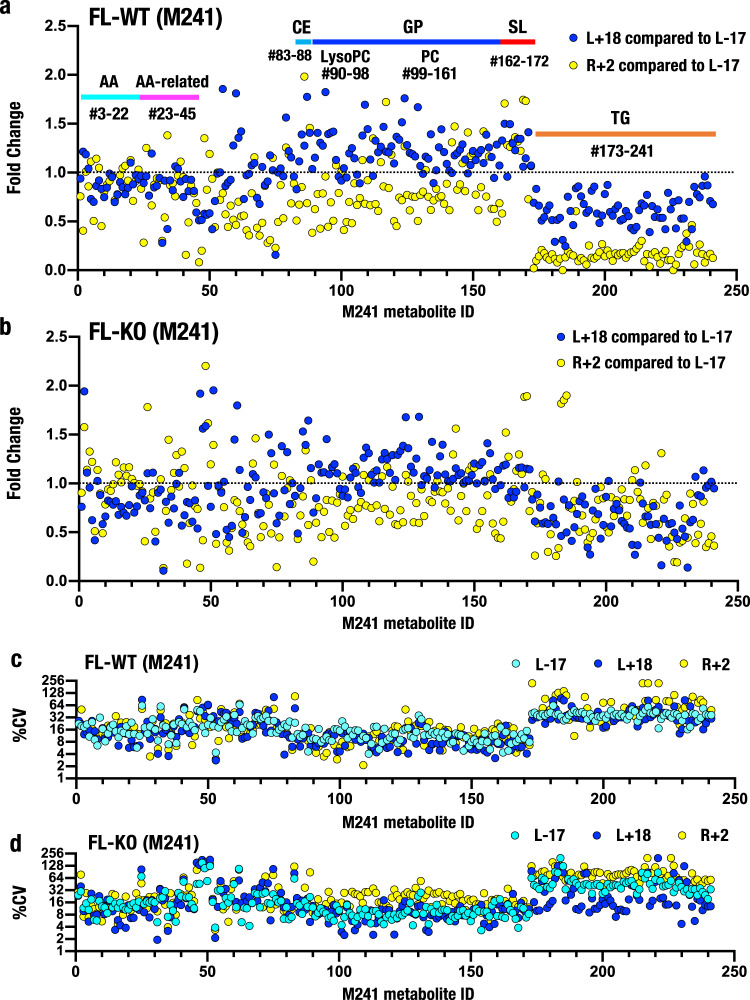
Plasma metabolite levels measured during and after spaceflight. **a**, **b** Metabolic changes in plasma collected from the tails of WT (**a**) and Nrf2 KO (**b**) mice (dataset M241). Fold changes in plasma metabolite levels at L+18 (blue dots) compared to L-17 and R+2 (yellow dots) compared to L-17. **c**, **d** CV values of dataset M241 in the plasma of WT (**c**) and Nrf2 KO (**d**) mice at three time points: WT L-17 (cyan), L+18 (blue) and R+2 (yellow) and Nrf2 KO L-17 (cyan), L+18 (blue) and R+2 (yellow). Data are presented as the CV (%) of the metabolite concentrations. The horizontal axis indicates M241 metabolite IDs (Supplementary Data 2) and includes 20 amino acids (#3–22), 23 amino acid-related metabolites (#23–45), 6 cholesteryl esters (#83–88), 9 lysophosphatidylcholines (#90–98), 63 phosphatidylcholines (#99–161), 11 sphingolipids (#162–172) and 69 triacylglycerols (#173–241).

The M104 dataset did not include triacylglycerol data, as triacylglycerol levels showed large individual differences. In contrast, dataset M241 includes 69 triacylglycerols (Supplementary Data 1). Therefore, we plotted relative metabolite levels according to metabolite ID. Showing very good agreement with the M104 heatmap analyses, the levels of glycerophospholipids (M241 ID #90–161) and sphingolipids (#162–172) in FL-WT mouse samples increased at L+18 compared to L-17 (Fig. 2a, blue dots). In contrast, glycerophospholipid levels were markedly reduced at R+2 (yellow dots). Triacylglycerol levels (#173–241) were reduced in the FL-WT group at L+18 and substantially decreased at R+2 compared with L-17, indicating that the triacylglycerol levels decreased during and after spaceflight.

Importantly, these changes in glycerophospholipid and triacylglycerol levels were markedly attenuated in the Nrf2 KO mouse plasma samples (Fig. 2b). The changes observed at L+18 (blue dots) and R+2 (yellow dots) were both substantially dampened in the Nrf2 KO mice. These results support our hypothesis that Nrf2 plays important roles in regulating triacylglycerol levels during spaceflight. Based on these collective results, we propose that Nrf2 reduces large-magnitude changes in metabolic homeostasis during spaceflight.

When we plotted the CV values of each metabolite *vs*. M241 ID, we observed higher CV values for triacylglycerols and lower CV values for cholesteryl esters, glycerophospholipids, and sphingolipids in both the FL-WT (Fig. 2c) and FL-KO groups (Fig. 2d), showing that this trend was quite reproducible. The explanation for the high CV values for triacylglycerols remains to be clarified.

### Identification of altered metabolite levels during spaceflight

We first performed principal component analysis (PCA) for a dimensional reduction of the M241 dataset to examine the effect of spaceflight on metabolic regulation. PC1 or PC2 did not separate the three time points L-17, L+18 and R+2 in GC-WT mice (Supplementary Fig. 2a). In contrast, PC1 separated R+2 and L-17, and PC2 separated L+18 and L-17 in FL-WT mice (Supplementary Fig. 2b). As shown in Fig. 1c, since the levels of glycerophospholipids were altered during spaceflight, we performed PCA of 72 glycerophospholipids. PC2 only slightly separated R+2, but not L+18, from the L-17 time point in GC-WT mice (Supplementary Fig. 2c). In contrast, both PC1 and PC2 separated L+18 and R+2 from L-17 in FL-WT mice (Supplementary Fig. 2d). Based on these results, spaceflight potently influenced plasma metabolite levels, and glycerophospholipid levels were altered in in-flight samples collected from FL-WT mice at L+18.

**Fig. 3:**
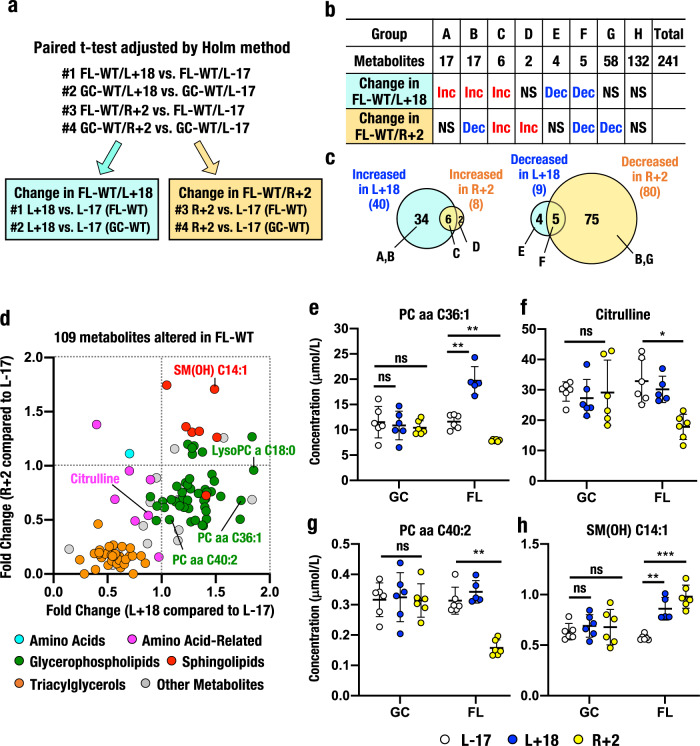
Metabolic responses observed in WT mice during and after spaceflight. **a** Statistical analyses to identify altered metabolites in FL-WT mice. Paired *t*-tests were performed between time points (L+18 *vs*. L-17, #1 for FL-WT and #2 for GC-WT; R+2 *vs*. L-17, #3 for FL-WT and #4 for GC-WT), and *P* values were adjusted using the Holm method. Altered metabolites were determined based on an adjusted *P* < 0.05 and combinations of comparisons (#1 and #2 for L+18, blue box; #3 and #4 for R+2, yellow box). **b** Numbers of increased (Inc) or decreased (Dec) metabolites during (change in FL-WT/L+18) and after (change in FL-WT/R+2) spaceflight. **c** Venn diagram of increased (left) or decreased (right) metabolites selected through a combination of statistical analyses. **d** Relationship between fold changes (FC) observed at L+18 compared to L-17 and R+2 compared to L-17 in FL-WT mice. The plot shows 109 metabolites whose levels were altered in FL-WT mice at L+18 (horizontal axis) and R+2 (vertical axis). Note the marked clusters of changes in metabolites in this plot. **e**–**h** Representative metabolites whose levels were altered during and after spaceflight (FL) compared with GC. One significantly altered metabolite at FL L+18 is PC aa C36:1 (**e**), two significantly altered metabolites at FL R+2 are citrulline (**f**) and PC aa C40:2 (**g**), and one significantly altered metabolite at both FL L+18 and R+2 is SM(OH) C14:1 (**h**). The results are presented as the mean plasma concentrations (μmol/L) ± SD. *Adjusted *P* < 0.05, **adjusted *P* < 0.01 and ***adjusted *P* < 0.001. ns not significant.

We next compared plasma metabolite levels between four pairs of time points: #1 L+18 *vs*. L-17 in FL-WT mice, #2 L+18 *vs*. L-17 in GC-WT mice, #3 R+2 *vs*. L-17 in FL-WT mice and #4 R+2 *vs*. L-17 in GC-WT mice (Fig. 3a, Supplementary Fig. 3a and Supplementary Data 2). We also compared the metabolite levels in FL-WT mice between two pairs of time points, L+18 *vs*. L-17 (Fig. 3a, blue box, #1) and R+2 *vs*. L-17 (yellow box, #3), to identify metabolites that were altered at L+18 (in-flight, blue box) and at R+2 (after return, yellow box) and to explore differences in the level of metabolites in the same mice during and after space travel. We also compared the level of metabolites in GC-WT mice between two pairs of time points, i.e., L+18 *vs*. L-17 (blue box, #2) and R+2 *vs*. L-17 (yellow box, #4), to exclude metabolites whose levels were not affected by spaceflight.

As shown in Supplementary Fig. 3a, b, plasma LysoPC C18:0 levels were significantly increased at L+18 in FL-WT mice compared with those measured at L-17 in FL-WT mice (#1, purple line). In contrast, the levels were comparable between L+18 and L-17 in GC-WT mice (#2, red line), indicating that the LysoPC C18:0 level was specifically increased during spaceflight. Meanwhile, as shown in Supplementary Fig. 3c, the LysoPC C18:0 levels measured at R+2 in FL-WT mice were comparable to those measured at L-17 in the same FL-WT mice (#3, purple line). Similarly, the level recorded at R+2 was comparable to that detected at L-17 in GC-WT mice (#4, red line). Thus, the plasma LysoPC C18:0 levels were increased specifically during spaceflight (L+18), but the levels were restored to control levels soon after return to the ground (R+2), indicating the importance of collecting plasma samples during spaceflight to clarify the genuine effects of space stress.

Based on these comparisons, we selected 109 metabolites that were significantly altered in the L+18 and R+2 samples from FL-WT mice for comparison with L-17 samples from FL-WT mice (#1 and #3, respectively). Precise changes are presented in Fig. 3b, Groups A–G. Metabolites that changed only at one time point are also shown in the panel; for example, when a metabolite level was increased in FL-WT/L+18 but not significantly altered in FL-WT/R+2 compared to FL-WT/L-17, the metabolite was categorized in Group A. Group H represents unchanged metabolites. These 109 modulated metabolites included 1 amino acid, 7 amino acid-related metabolites, 44 glycerophospholipids, 7 sphingolipids, 36 triacylglycerols and 14 other metabolites (Supplementary Fig. 3d and Supplementary Data 2).

Of the 109 metabolites, 40 (Fig. 3b, A–C) and 8 (C and D) metabolite levels were elevated in FL-WT mice at L+18 and R+2 compared to L-17, respectively, indicating that the levels of a considerable number of metabolites were indeed increased during spaceflight. Venn diagrams illustrate that the levels of 34 metabolites (Fig. 3c, Groups A and B) were increased only at L+18, while the levels of 2 metabolites (D) were increased only at R+2, and the levels of 6 metabolites (C) were commonly increased at both L+18 and R+2 (left). In clear contrast, the levels of 9 (Fig. 3b, Groups E and F) and 80 (Groups B, F and G) metabolites were decreased at L+18 and R+2 compared to L-17 in FL-WT mice, respectively. These observations suggest that space stresses promote increased levels of plasma metabolites, but the return to Earth gravity acts to swiftly reduce the levels of these metabolites, e.g., PC aa C36:1 (Fig. 3e).

We evaluated the relationships between in-flight (L+18) and post-flight (R+2) changes in metabolite levels in FL-WT mice by plotting fold changes (FCs) at L+18 and R+2 *vs*. L-17 (Fig. 3d). The metabolite that showed the greatest increase at L+18 was LysoPC a C18:0, the details of which are shown in Supplementary Fig. 3b, c. Notably, the levels of many glycerophospholipids (green dots) were increased at L+18 but decreased at R+2. In contrast, the levels of several sphingolipids (red dots) were increased at both L+18 and R+2, and the sphingolipid with the highest level measured at R+2 was SM(OH) C14:1. The levels of an amino acid (cyan plot) and amino acid-related metabolites (magenta plots) were decreased at both L+18 and R+2. Most impressively, triacylglycerol levels (orange plots) were markedly decreased at both L+18 and R+2. The explanation for why these groups of metabolites showed distinctly different profiles remains to be clarified.

Two representative metabolites that showed significant alterations at L+18 (in-flight) were LysoPC a C18:0 (Supplementary Fig. 3b) and PC aa C36:1 (Fig. 3e). Plasma levels of LysoPC a C18:0 and PC aa C36:1 were increased in FL-WT mice at L+18. In contrast, the levels of these metabolite did not change substantially in GC-WT mice.

Three representative metabolites whose levels did not change substantially during spaceflight but showed significant alterations after return to Earth (at R+2) were citrulline and PC aa C40:2 (Fig. 3f, g). The levels of these metabolites were markedly decreased in the post-flight (R+2) samples from FL-WT mice; a similar decrease was not observed in GC-WT mice. In contrast, the plasma levels of SM(OH) C14:1 were increased (Fig. 3h) at both L+18 and R+2 in FL-WT mice, but the levels did not change substantially at the three time points in GC-WT mice. Thus, the metabolic response during spaceflight involves diverse metabolites but is predominantly characterized by increases in glycerophospholipid and sphingolipid levels, as well as decreases in triacylglycerol levels.

### Nrf2 contributes to metabolic regulation in mice during spaceflight

**Fig. 4:**
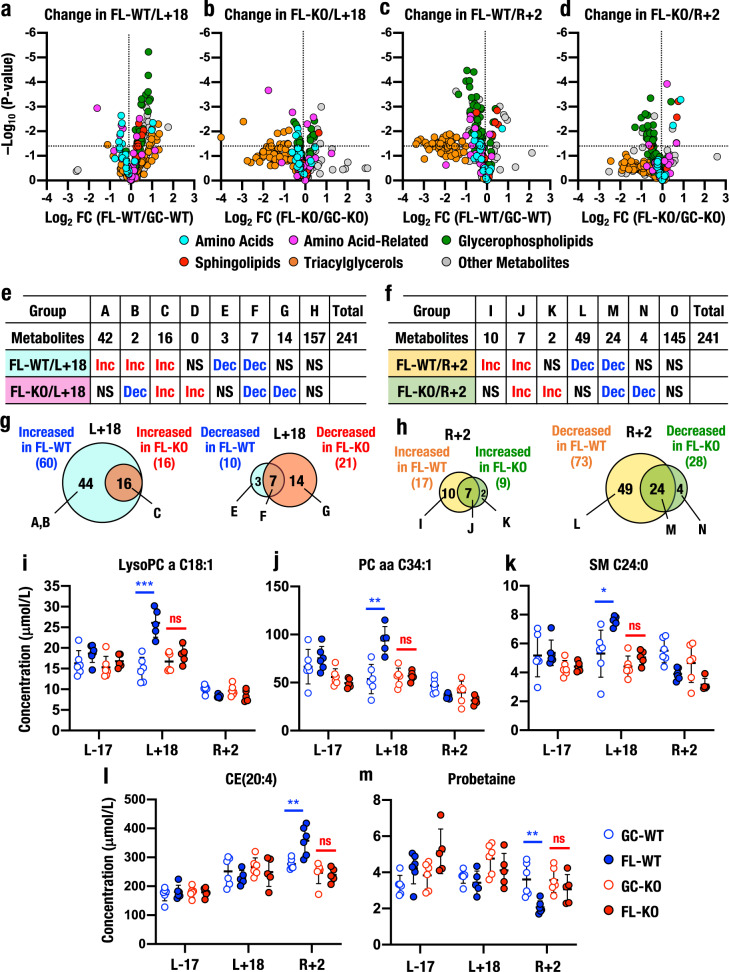
Role of Nrf2 in metabolic changes during and after spaceflight. **a**–**d** Volcano plots. The x-axis shows the log_2_ fold change (FC) of plasma levels in FL-WT relative to GC-WT at L+18 (**a**) and R+2 (**c**), and FL-KO relative to GC-KO at L+18 (**b**) and R+2 (**d**). The y-axis shows the negative log_10_ of the two-tailed test *P* value. Vertical dotted lines denote a linear fold change of one. Horizontal dotted lines indicate *P* = 0.05. **e**–**h** Numbers of increased (Inc) or decreased (Dec) metabolites in FL-WT and FL-KO mice at L+18 (**e**) and R+2 (**f**). Venn diagram of changes at L+18 (**g**) and R+2 (**h**). Altered metabolites in FL-WT/L+18 (*vs*. GC-WT/L+18), FL-KO/L+18 (*vs*. GC-KO/L+18), FL-WT/R+2 (*vs*. GC-WT/R+2) and FL-KO/R+2 (*vs*. GC-KO/R+2) were determined based on an adjusted *P* < 0.05. **i**–**m** Representative metabolites that were regulated by Nrf2 during spaceflight. Three metabolites with significantly increased levels in FL-WT mice at L+18 that were abrogated in FL-KO mice at L+18 were LysoPC a C18:1 (**i**), PC aa C34:1 (**j**) and SM C24:0 (**k**). One metabolite with a significantly increased level in FL-WT mice at R+2 that was abrogated in FL-KO mice at R+2 was CE(20:4) (**l**), and another metabolite with a significantly decreased level in FL-WT mice at R+2 that was abrogated in FL-KO mice at R+2 was probetaine (**m**). The results are presented as the mean plasma concentrations (μmol/L) ± SD. Statistical analyses were performed using a *t*-test adjusted using the Holm method. *Adjusted *P* < 0.05, **adjusted *P* < 0.01 and ***adjusted *P* < 0.001.

We clarified whether Nrf2 contributes to metabolic regulation during and after spaceflight by conducting metabolome analyses of plasma samples obtained from Nrf2 KO mice using similar protocols. The volcano plots of dataset M241 revealed that the levels of many metabolites, including triacylglycerols, glycerophospholipids and amino acid-related metabolites, were increased in FL-WT mice compared to GC-WT mice at L+18 (Fig. 4a), but the levels of many of the metabolites, especially triacylglycerols and amino acids, were lower in Nrf2 KO mice during spaceflight (FL-KO) than in GC-KO mice (Fig. 4b). Most impressively, the changes in glycerophospholipid levels detected in FL-WT mice were not observed in FL-KO mice. Based on these results, Nrf2 KO substantially influences the changes in the levels of these metabolites elicited during spaceflight.

Meanwhile, after returning to ground (i.e., mice at R+2), the levels of many metabolites that had increased during spaceflight were decreased (Fig. 4c). Importantly, these changes were markedly abolished in FL-KO mice, and the number of metabolites that showed a statistically significant decrease was substantially suppressed in FL-KO mice compared with FL-WT mice (Fig. 4d). These results again indicate that metabolic responses during spaceflight were significantly different from those observed after the return to ground and that Nrf2 KO attenuates metabolic responses during and after spaceflight. These observations reinforce the hypothesis that the plasma analyses performed at three time points in this study provide insights into the changes in metabolic regulation that accompany space travel and return to ground.

### Identification of individual metabolites regulated by Nrf2

We next evaluated the changes in individual metabolites. For this experiment, we compared plasma metabolite levels in four pairs of GC *vs*. FL samples (#1 FL-WT *vs*. GC-WT at L+18; #2 FL-KO *vs*. GC-KO at L+18; #3 FL-WT *vs*. GC-WT at R+2; #4 FL-KO *vs*. GC-KO at R+2; Supplementary Fig. 4a and Supplementary Data 3). Of the 60 metabolites whose levels were increased in the FL-WT/L+18 group compared with the GC-WT/L+18 group (Fig. 4e, Groups A–C), the levels of 44 metabolites were not elevated in the FL-KO/L+18 group (Fig. 4e, Groups A and B and Supplementary Fig. 4b). The levels of the remaining 16 metabolites were increased in both FL-WT and FL-KO mice at L+18 (Group C). Moreover, while the levels of 10 metabolites were decreased in the FL-WT/L+18 group (Groups E and F), decreases in the levels of 3 of these metabolites were not observed in the FL-KO/L+18 group (Group E). These relationships are shown in a Venn diagram (Fig. 4g), and Nrf2 KO indeed significantly altered the increase in plasma metabolite levels during spaceflight.

As for the analysis of mice after the return to the ground, we were surprised to detect decreased levels of 73 metabolites in FL-WT mice at R+2 (Fig. 4f, Groups L and M, and Fig. 4h). The levels of 49 of these 73 metabolites did not decrease in FL-KO/R+2 mice. In contrast to the FL-WT/L+18 mice, the levels of 17 metabolites were increased in FL-WT/R+2 mice (Fig. 4f, Groups I and J), and the levels of 9 of these metabolites were elevated in FL-KO/R+2 mice (Groups J and K). Group I included 3 glycerophospholipids and 2 sphingolipids, and Group L included 22 glycerophospholipids and 15 triacylglycerols (Supplementary Fig. 4c).

An inspection of individual metabolite levels revealed that the plasma levels of LysoPC a C18:1 (Fig. 4i), PC aa C34:1 (Fig. 4j) and SM C24:0 (Fig. 4k) were significantly increased in FL-WT/L+18 samples compared to the GC-WT/L+18 samples. The plasma levels of CE(20:4) were increased (Fig. 4l), but those of probetaine were decreased (Fig. 4m) in FL-WT/R+2 mice compared to GC-WT/R+2 mice. These changes were not observed in FL-KO mice, indicating that Nrf2 contributes to the changes in plasma glycerophospholipid and sphingolipid levels at L+18 and triacylglycerol levels at R+2. These results further support our hypothesis that Nrf2 plays critical roles in metabolic responses during spaceflight.

### Regulation of lipid metabolism in eWAT

We previously identified that the size of eWAT lipid droplets increases significantly during spaceflight^24^. We assessed the relationships between lipid droplet size in eWAT and metabolic changes in the plasma in this study by conducting lipidomics of eWAT samples collected at R+2. For this analysis, we selected 60 lipids using a criterion of a CV of 30% or less. These lipids included 8 phosphatidylcholines and 52 triacylglycerols and are collectively referred to as dataset M60.

**Fig. 5:**
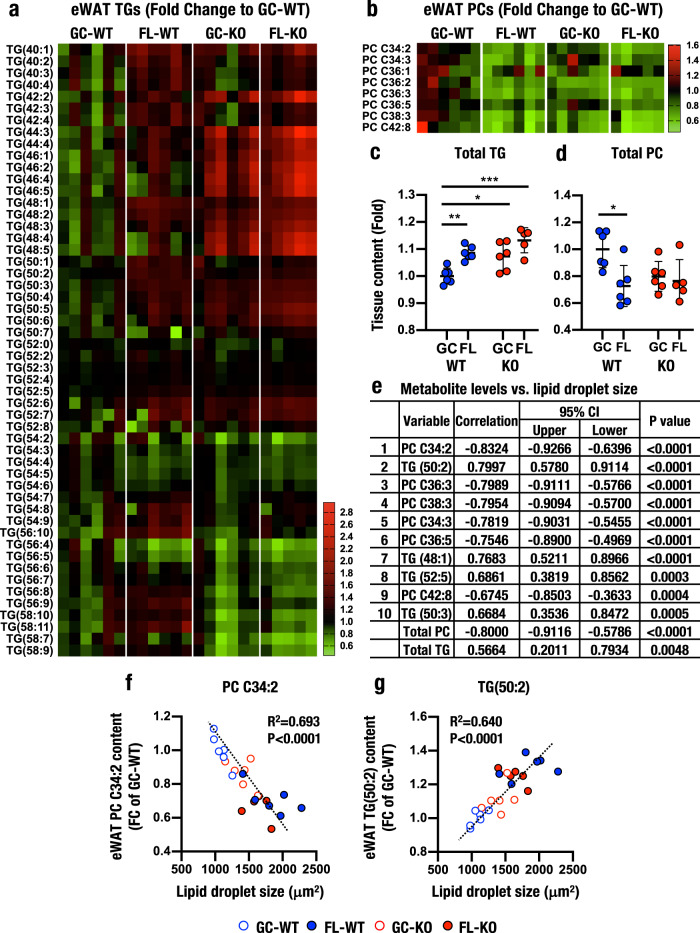
Lipid metabolism in eWAT. **a**, **b** Heatmap analyses of triacylglycerol (**a**) and phosphatidylcholine (**b**) levels in eWAT at R+2. The means of GC-WT/R+2 are set to one. **c**, **d** Total triacylglycerol (**c**) and phosphatidylcholine (**d**) levels in the eWAT of WT (blue) and Nrf2 KO (red) mice. The results are presented as the fold change compared to GC-WT mice and the means ± SD. Statistical analyses were performed using ANOVA followed by Tukey’s post hoc test. *P < 0.05, **P < 0.01 and ***P < 0.001. **e**–**g** Multivariate analysis (**e**) and levels of individual metabolites PC C34:2 (**f**) and TG (50:2) (**g**) to determine the relationship between lipid levels and the lipid droplet size in eWAT. Notably, the levels of PC C34:2 and TG (50:2) show very good correlations with the lipid droplet size. *P* values and Pearson’s correlation coefficient *R* (**e**) or *R*^2^ (**f**, **g**) values with the corresponding 95% confidence interval (95% CI) are displayed (**e**). The top 10 metabolites and total triacylglycerol and phosphatidylcholine levels are indicated (**e**).

The heatmap of M60 revealed that triacylglycerol levels were broadly and markedly increased in the eWAT of FL-WT/R+2 compared to that of GC-WT/R+2 (Fig. 5a). The levels of these triacylglycerols were increased even more significantly in the eWAT of GC-KO/R+2 and FL-KO/R+2 mice. In particular, the levels of triacylglycerols with lengths ranging from 42:2 to 48:5 were markedly elevated in both GC-KO/R+2 and FL-KO/R+2 eWAT. The levels of triacylglycerols with lengths ranging from 50:1 to 50:6, 54:6 to 56:10 and 56:9 to 58:11 were also increased in FL-WT/R+2 eWAT. In contrast, levels of longer triacylglycerols ranging from 54:6 to 56:10 and 56:9 to 58:11 were clearly decreased in both GC-KO/R+2 and FL-KO/R+2 eWAT. Based on these results, Nrf2 deficiency induced the accumulation of shorter triacylglycerols and decreased the levels of longer triacylglycerols in eWAT both during space travel and on the ground.

We also evaluated the levels of 8 phosphatidylcholines. The levels of these phosphatidylcholines were decreased in FL-WT/R+2, GC-KO/R+2 and FL-KO/R+2 eWAT compared to GC-WT/R+2 eWAT (Fig. 5b). Importantly, space travel and the Nrf2 KO genotype induced similar changes in the levels of these phosphatidylcholines but did not provoke strong additive changes. We then evaluated the total triacylglycerol and phosphatidylcholine levels in the eWAT of all mice exposed to spaceflight and found that total triacylglycerol levels were elevated in both FL-WT/R+2 and GC-KO/R+2 eWAT (Fig. 5c). Total phosphatidylcholine levels decreased in the FL-WT/R+2, GC-KO/R+2 and FL-KO/R+2 eWAT compared to GC-WT/R+2 eWAT (Fig. 5d).

We also examined the levels of individual triacylglycerols and phosphatidylcholines in the eWAT (Supplementary Fig. 5). TG(40:2) levels were increased in FL-WT eWAT compared to GC-WT/R+2 eWAT, but the increased was blunted by Nrf2 KO (panel a). TG(50:4) and TG(50:5) levels increased in both FL-WT and GC-KO eWAT, and the levels were additively increased in FL-KO eWAT (panels b and c). The levels of TG(44:3), TG(46:4) and TG(48:5) were markedly increased in both GC-KO/R+2 and FL-KO/R+2, and the levels were comparable between GC-KO and FL-KO/R+2 (panels d to f). The levels of PC C34:2, PC C36:2 and PC C38:3 decreased in FL-WT eWAT compared to GC-WT eWAT. The levels of these metabolites also decreased reproducibly in GC-KO and FL-KO eWATs (panels g to i). Therefore, space travel and the Nrf2 KO genotype additively and independently affect the levels of these metabolites in eWAT, depending upon the nature of the metabolites.

We next performed a multivariate analysis of the relationship between the eWAT lipid levels in dataset M60 and lipid droplet size and determined the top 10 metabolites that contributed to droplet size. These 10 metabolites included 6 phosphatidylcholines and 4 triacylglycerols; the former correlated negatively, whereas the latter correlated positively with the lipid droplet size (Fig. 5e). The results for representative metabolites, PC C34:2 and TG(50:2), are shown in Fig. 5f, g, respectively. Lipid droplet size was strongly correlated with the levels of PC C34:2 and TG(50:2) in eWAT. These data indicate that both spaceflight and the Nrf2 KO genotype modulated the triacylglycerol and phosphatidylcholine contents, which correlated with eWAT lipid droplet size. Thus, we conclude that Nrf2 contributes to the regulation of lipid metabolism in eWAT both on the ground and in space.

### RNA-seq analyses of eWAT, liver and the cerebrum

**Fig. 6:**
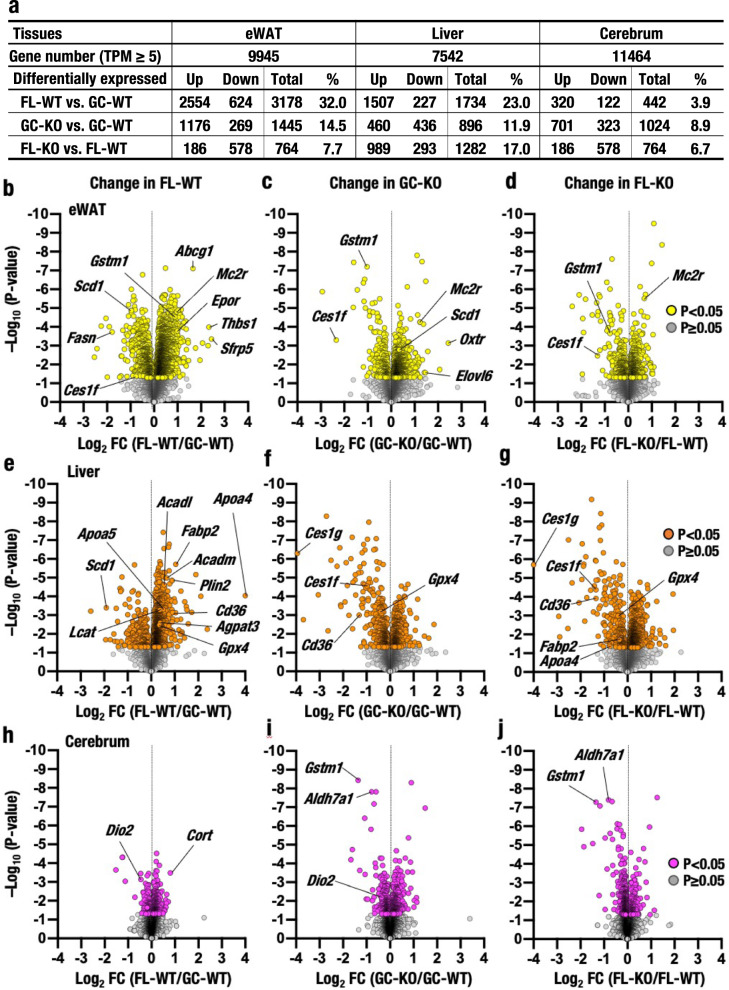
RNA-seq analyses of the eWAT, liver and cerebrum. **a** Numbers of total genes (mean TPM value ≥5) and differentially expressed genes (*P* < 0.05 according to the two-tailed test) identified in RNA-seq analyses of the eWAT, liver and cerebrum of GC-WT (*n* = 6), FL-WT (*n* = 6), GC-KO (*n* = 6) and FL-KO (*n* = 5) mice. **b**–**j** Volcano plots of RNA-seq analyses. The x-axis represents the log_2_ fold change (FC) in the eWAT (**b**–**d**), liver (**e**–**g**) and cerebrum (**h**–**j**) of FL-WT mice compared to GC-WT mice (**b**, **e**, **h**), GC-KO mice compared to GC-WT mice (**c**, **f**, **i**) and FL-KO mice compared to FL-WT mice (**d**, **g**, **j**). The y-axis represents the negative log_10_ of the two-tailed *P* value. Vertical dotted lines denote a fold change of one. Yellow (**b**–**d**), magenta (**e**–**g**) and orange (**h**–**j**) dots denote *P* < 0.05, and grey dots denote *P* ≥ 0.05 (**b**–**j**).

We examined the mechanisms underlying the changes in the metabolome and gene expression during spaceflight by conducting RNA-seq analyses. For the RNA-seq analyses, we selected the liver, cerebrum and eWAT because of the novel contributions of these organs and tissue to the regulation of lipid metabolism. With a mean transcripts per million (TPM) value ≥5 as the selection criterion, we selected 9945, 7542 and 11464 genes in eWAT, liver and cerebrum, respectively (Fig. 6a and Supplementary Data 4-6). We evaluated differentially expressed genes in space-travel mice *vs*. ground control mice and found that 32.0%, 23.0% and 3.9% of genes were altered in these three tissues/organs from FL-WT/R+2 and GC-WT/R+2 mice, respectively.

We further evaluated how the loss of Nrf2 influenced these changes by examining the tissues from Nrf2 KO mice and found that the expression of 14.5% and 7.7% of genes was altered in GC-KO/R+2 and FL-KO/R+2 eWAT samples compared with GC-WT/R+2 and FL-WT/R+2 eWAT, respectively. Similarly, we observed altered expression of 11.9% and 17.0% genes in the liver and 8.9% and 6.7% genes in the cerebrum of GC-KO/R+2 and FL-KO/R+2 mice compared with GC-WT/R+2 and FL-WT/R+2 mice, respectively (Fig. 6a). Thus, spaceflight itself influenced gene expression more substantially than Nrf2 KO in the eWAT and liver. In contrast, spaceflight modulated gene expression in the cerebrum less significantly than the Nrf2 KO genotype.

We then constructed volcano plots of the gene expression data from eWAT (Fig. 6b–d), liver (Fig. 6e–g) and cerebrum (Fig. 6h–j). Notably, while space travel and Nrf2 KO both broadly affected the gene expression profiles in eWAT (Fig. 6b and c, respectively), the differences in the profiles between FL-KO/R+2 and FL-WT/R+2 eWAT were rather smaller (Fig. 6d) than those in the FL-WT/R+2 and GC-WT/R+2 eWAT (Fig. 6b) and GC-KO/R+2 and GC-WT/R+2 eWAT (Fig. 6c) comparisons, suggesting that the space stresses and Nrf2 KO appeared to counteract the effects of the other on changing in gene expression. In contrast, while space travel and Nrf2 KO widely affected the gene expression profile in the liver (Fig. 6e and f, respectively), the difference between FL-KO/R+2 and FL-WT/R+2 livers (Fig. 6g) was significantly larger than that in GC-KO/R+2 and GC-WT/R+2 livers (Fig. 6f), suggesting that space stresses augmented the changes in gene expression observed in Nrf2 KO mouse livers.

We did not observe substantial changes in gene expression in the cerebrum induced by space stresses or Nrf2 KO (Fig. 6h–j). These results support our hypothesis that space stresses activate multiple and complex tissue- and organ-specific signalling pathways and that the Nrf2 pathway is an important pathway that responds to space stresses in various tissues and organs.

### Expression of individual metabolism-related genes in eWAT after space travel

**Fig. 7:**
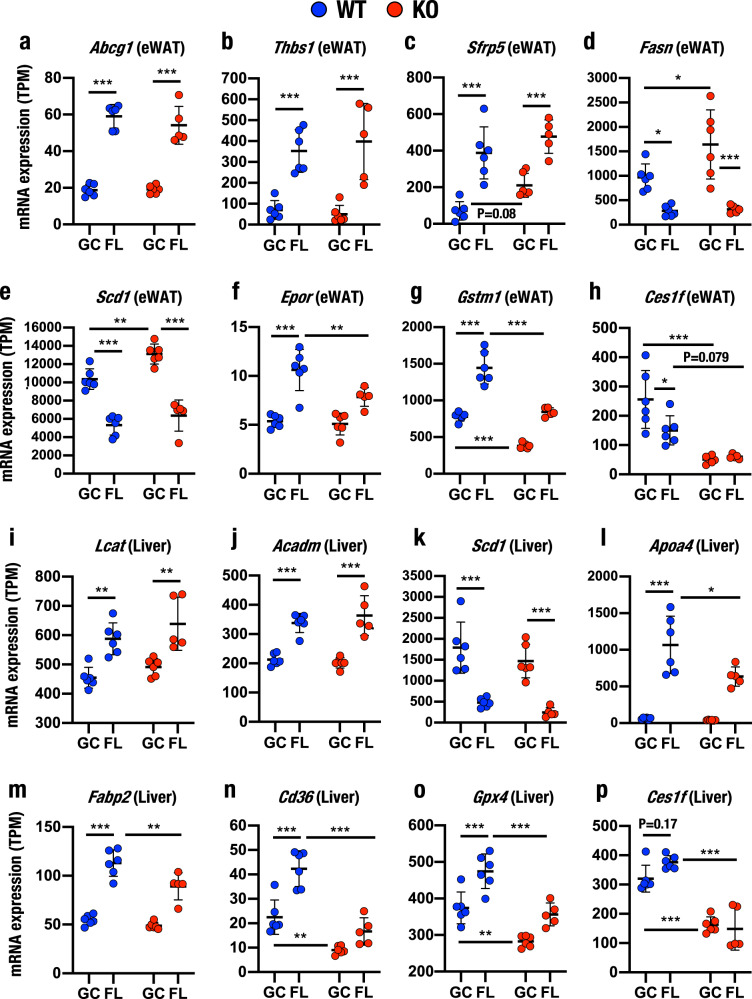
Differentially expressed genes identified in the eWAT and liver after spaceflight. **a**–**p** Expression levels of representative differentially expressed genes in eWAT (**a**–**h**) and liver (**i**–**p**) determined using RNA-seq analyses. Expression levels (TPM) of *Abcg1* (**a**), *Thbs1* (**b**), *Sfrp5* (**c**), *Fasn* (**d**), *Scd1* (**e**, **k**), *Epor* (**f**), *Gstm1* (**g**), *Ces1f* (**h**, **p**), *Lcat* (**i**), *Acadm* (**j**), *Apoa4* (**l**), *Fabp2* (**m**), *Cd36* (**n**) and *Gpx4* (**o**) are presented as the means ± SD. Statistical analyses were performed using ANOVA followed by Tukey’s post hoc test. *P < 0.05, **P < 0.01 and ***P < 0.001.

We next explored the changes in the expression of representative metabolism-related genes in the eWAT, liver and cerebrum. We selected representative genes by analysing volcano plots. We first examined eWAT and selected 11 genes, including *Abcg1* and *Thbs1*, which contribute to lipid storage in adipocytes; *Sfrp5*, an adipokine contributing to regulating lipid metabolism; *Ces1f*, which contributes to triacylglycerol hydrolysis; and *Fasn*, *Scd1* and *Elovl6*, which play important roles in FA synthesis (Supplementary Data 7). These genes encode important enzymes and transporters that are related to lipid metabolism. Of the remaining genes, *Epor*, *Mc2r* and *Oxtr* encode hormone receptors, and *Gstm1* plays critical roles in detoxification and antioxidant functions. Of these 11 genes, the expression levels of *Abcg1*, *Thbs1*, *Sfrp5*, *Epor*, *Gstm1*, *Mc2r* and *Oxtr* were elevated in FL-WT/R+2 eWAT compared to GC-WT/R+2 eWAT. In contrast, the expression of *Fasn*, *Scd1* and *Ces1f* decreased and that of *Elovl6* was not altered in FL-WT/R+2 eWAT compared to GC-WT/R+2 eWAT (Fig. 7a–h and Supplementary Fig. 6a-c).

We next evaluated how the loss of Nrf2 altered the expression of these 11 genes. Based upon the responses to space stresses and Nrf2 KO, we classified the genes into three categories. First, we observed comparable expression of *Abcg1* and *Thbs1* between GC-WT/R+2 and GC-KO/R+2 and between FL-WT/R+2 and FL-KO/R+2, indicating that space stresses induced the expression of these two genes through certain signalling pathway(s) independent of the Nrf2 signalling pathway. We named this category as Class 1.

Second, the expression of *Srfp5*, *Fasn*, *Scd1* and *Elovl6* was significantly induced in the GC-KO/R+2 eWAT compared with that of GC-WT/R+2 eWAT (Fig. 7c–e, Supplementary Fig. 6a). Of these four genes, *Srfp5* expression increased, but the expression of the other three genes decreased in the FL-KO/R+2 eWAT compared with GC-KO/R+2 eWAT. Importantly, the expression of these four genes was comparable between FL-WT/R+2 and FL-KO/R+2 eWAT. These results thus indicate that Nrf2 KO-induced changes in the expression of these genes were either abrogated or attenuated by exposure to space stresses. Certain signalling pathway(s) regulate gene expression during spaceflight, and the effect of spaceflight seems to be stronger than that of the Nrf2 signalling pathway, such that space stresses either override or counteract the regulatory cue from Nrf2 KO. We named this category of genes as Class 2.

In the third category of genes, or Class 3, we identified five genes for which Nrf2 KO dominated the effect of space stresses. Of these five genes, *Epor*, *Gstm1, Mc2r* and *Oxtr* were expressed at higher levels after exposure to space stresses, while *Ces1f* was expressed at a lower level. Nrf2 KO either abolished or substantially weakened their expression in eWAT (Fig. 7f–h and Supplementary Fig. 6b, c). The increase in *Epor* and *Gstm1* expression observed during spaceflight was substantially abrogated by Nrf2 KO. The expression of *Ces1f* was strongly repressed by Nrf2 KO, while that of *Mc2r* was increased substantially in both GC-KO/R+2 and FL-KO/R+2 eWAT. Thus, while the expression of these genes was regulated by both Nrf2 and space stresses, Nrf2 signalling appeared to dominate the regulatory mechanism.

### Expression of metabolism-related genes in the liver

We next evaluated the hepatic expression of a selection of 13 genes that showed marked changes in the volcano plots. These genes included *Lcat*, *Acadm* and *Acadl*, *Apoa4* and *Apoa5*, *Fabp2* and *Cd36*, all of which contribute to lipid metabolism (Supplementary Data 7). *Plin2* plays important roles in lipid droplet formation, while *Ces1f* and *Ces1g* regulate triacylglycerol hydrolysis as described above. The expression levels of the *Lcat*, *Acadm*, *Apoa4*, *Fabp2*, *Cd36*, *Gpx4*, *Acadl*, *Apoa5*, *Plin2* and *Agpat3* genes were increased in FL-WT/R+2 livers compared with GC-WT/R+2 livers. In contrast, *Scd1* expression was decreased in the livers of FL-WT/R+2, and *Ces1f* and *Ces1g* were expressed at comparable levels between GC-WT/R+2 and FL-WT/R+2 livers (Fig. 7i–p, Supplementary Fig. 6d-h).

Similar to the analyses performed in eWAT, we analysed the effect of Nrf2 deletion and found that the expression of the *Lcat*, *Acadm*, *Acadl*, *Scd1*, *Apoa5*, *Plin2* and *Agpat3* genes was not altered in FL-KO/R+2 livers compared to FL-WT/R+2 livers (Fig. 7i–k and Supplementary Fig. 6d–g). The expression of these genes in the liver was also comparable between GC-WT/R+2 and GC-KO/R+2, indicating that space stresses induced the expression of these seven genes independent of the Nrf2 signalling pathway and that these genes belong to Class 1.

In contrast, the expression levels of *Apoa4*, *Fabp2*, *Cd36*, *Gpx4* and *Ces1f* were significantly suppressed in FL-KO/R+2 livers compared to FL-WT/R+2 livers (Fig. 7l–p). Although *Apoa4* and *Fabp2* expression levels in the GC-KO/R+2 liver were comparable to those in the GC-WT/R+2 liver, the levels of *Cd36*, *Gpx4* and *Ces1f* were decreased in the GC-KO/R+2 liver compared to the GC-WT/R+2 liver. Thus, Nrf2 signalling appeared to dominate the regulation of these five genes, indicating that these genes belong to Class 3. In addition, the expression of *Ces1g* was markedly suppressed in both GC-KO/R+2 and FL-KO/R+2 livers (Supplementary Fig. 6h).

These results thus support our contention that Nrf2 contributes to the expression of a wider range of genes in the liver than it does in eWAT during spaceflight and that space stresses and Nrf2 appear to collaboratively and individually modulate gene expression, depending upon the genes and tissues/organs.

### Gene expression in the cerebrum after spaceflight

Finally, we assessed changes in gene expression in the cerebrum after spaceflight. Similar to the eWAT and liver analyses, we aimed to select genes that showed significant changes during spaceflight and/or in the Nrf2 KO genotype by constructing volcano plots; however, we observed far fewer changed genes in the cerebrum than in the eWAT or liver (Fig. 6h–j). Ultimately, we selected the *Cort*, *Dio2*, *Gstm1* and *Aldh7a1* genes in the cerebrum (Supplementary Data 7). *Cort* expression was increased while *Dio2* expression was decreased in the cerebrum after spaceflight (comparison of FL-WT/R+2 with GC-WT/R+2; Supplementary Fig. 6i,j). The expression levels of the *Gstm1* and *Aldh7a1* genes did not change substantially between the GC-WT/R+2 and FL-WT/R+2 cerebrum (Supplementary Fig. 6k, l).

The expression of the *Cort* gene in the cerebrum was not influenced by Nrf2 KO (i.e., the levels were comparable between FL-WT/R+2 and FL-KO/R+2; Supplementary Fig. 6i). In stark contrast, the expression levels of *Dio2*, *Gstm1* and *Aldh7a1* in the cerebrum were substantially reduced by Nrf2 KO (GC-KO/R+2 and FL-KO/R+2 *vs*. GC-WT/R+2 and FL-WT/R+2; Supplementary Fig. 6j-l). Thus, we did not identify any case of cooperative regulation of gene expression by Nrf2 or other signalling pathways in the cerebrum during spaceflight.

### Decreased phosphatidylcholine levels in the brain after spaceflight

While we found that space stress altered the expression of fewer genes in the cerebrum than in the eWAT and liver, this result might reflect the use of the cerebrum *en bloc* in the analysis. In fact, spaceflight perturbs functions of the central nervous system^32^; moreover, the brain contains abundant phospholipids^33^, and glycerophospholipids are differentially distributed in various regions of the brain^34^. Therefore, we decided to examine the phosphatidylcholine levels in the brain using MALDI-MSI technology.

In Supplementary Fig. 7a, b, we present representative images of PC C34:1 and C36:4, respectively. Notably, we observed a marked decrease in the PC C34:1 signal in MSI in the cortex (Cor), hippocampus (Hipp) and thalamus (Thal) samples from FL-WT/R+2 and FL-KO/R+2 mice compared to GC-WT/R+2 and GC-KO/R+2 mice. Similar changes were observed for PC C36:4. When the MSI signals were quantified, we found that the PC C34:1 and C36:4 signals in FL-WT/R+2 mice were slightly lower than those in GC-WT/R+2 mice in both the cortex and hippocampus (Supplementary Fig. 7c, d). Importantly, this decrease was significant in FL-KO/R+2 mice compared with GC-KO/R+2 mice, indicating that the decrease after spaceflight occurred in the absence of Nrf2.

We then analysed the intensity of the MSI signals for many phosphatidylcholine metabolites and presented heatmaps for the six highest MSI signals, namely, C32:0, C34:1, C36:1, C36:4, C38:4 and C38:6. All signal intensities were decreased in the cortex and hippocampus (Supplementary Fig. 7e, f) of mice exposed to spaceflight (i.e., FL-WT/R+2 and FL-KO/R+2). The levels of these phosphatidylcholines did not decrease in GC-KO/R+2 samples compared with GC-WT/R+2 samples. According to these results, after space travel, the levels of a set of phosphatidylcholines decrease significantly in the brain, but these changes were not detected at the level of gene expression. The Nrf2 KO genotype did not exacerbate the decreases in the levels of these phosphatidylcholines, suggesting that these decreases occurred independently of the loss of Nrf2 activity.

## Discussion

We examined how space stress or the loss of Nrf2 influences metabolic regulation in mice individually and in combination by conducting detailed metabolome analyses utilizing plasma samples collected at pre-flight, in-flight and post-flight time points in space-travelled and ground control mice in the MHU-3 project^24^. As shown in the NASA Twins Study, blood levels of inflammatory markers are markedly increased upon the return of the astronaut to Earth gravity^3,35^. Therefore, we surmise that post-flight studies may reflect the combination of genuine space stresses and the effects of returning to Earth gravity. To the best of our knowledge, this study is the first to successfully complete a three-time point comparison of plasma metabolites using blood samples collected from the tail before, during and after spaceflight. Since the in-flight samples obtained in this study are not impacted by re-entry to gravity, this study permits us to evaluate the effect of space stresses independently of the stresses of returning to Earth gravity.

**Fig. 8:**
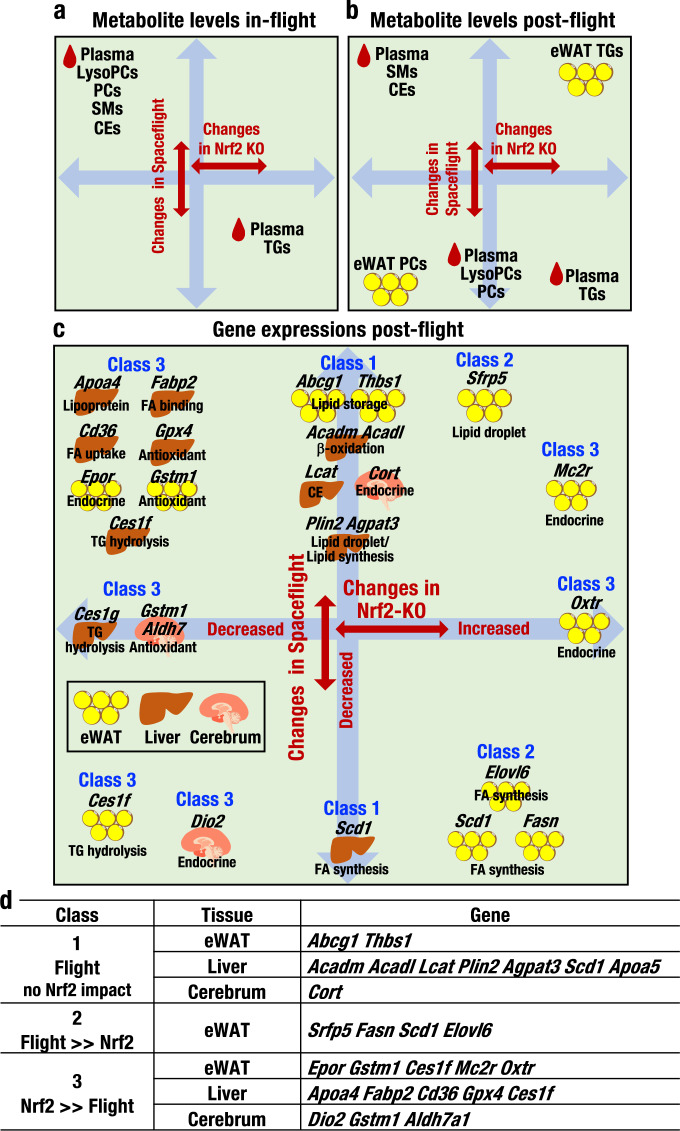
Metabolic response and changes in gene expression during and after spaceflight. **a**–**c** Schematic representations of metabolic responses during (**a**) and after (**b**) spaceflight. Vertical arrows denote changes in metabolite levels in the plasma and eWAT during (**a**) or after (**b**) spaceflight. Horizontal arrows denote these changes in Nrf2 KO mice. Schematic representations of changes in gene expression in the eWAT, liver and cerebrum after spaceflight are also provided (**c**). Vertical arrows denote changes in gene expression levels in the eWAT, liver and cerebrum after spaceflight (**c**). Horizontal arrows denote these changes in Nrf2 KO mice. **d** Classes of genes in the eWAT, liver and cerebrum. Class 1 includes genes with substantially altered expression in response to spaceflight that are not influenced by the Nrf2 KO genotype; Class 2 includes genes whose expression is affected by Nrf2 KO, but the changes are overridden by spaceflight; and Class 3 includes genes that are modulated by spaceflight, but the changes are overridden by Nrf2 KO.

As summarized in Fig. 8a, the plasma levels of lysophosphatidylcholines, phosphatidylcholines, sphingomyelins and cholesteryl esters increased during spaceflight. In contrast, as summarized in Fig. 8b, plasma levels of triacylglycerols decreased after returning to Earth. Importantly, these metabolic changes in plasma lipid levels were largely abrogated in Nrf2 KO mice. Notably, in the tissue metabolome analyses using the spaceflight samples, triacylglycerol levels increased, while phosphatidylcholine levels decreased in eWAT (Fig. 8b), suggesting the presence of dynamic metabolic changes in plasma and tissues after returning to Earth gravity. Thus, plasma metabolite levels are substantially different between in-flight samples and post-flight samples and that Nrf2 activity in fact alters metabolite levels in these plasma samples.

We also conducted extensive transcriptome analyses utilizing tissues from post-flight and ground control mice. We categorized differentially expressed genes into three classes according to their responses to space stresses and loss of Nrf2. The expression of genes in Class 1 changed substantially in response to space travel but not Nrf2 KO. The expression of genes in Class 2 was influenced by Nrf2 KO but substantially overridden by space stresses, and the expression of genes in Class 3 was influenced by space travel, but the changes were largely overridden by the loss of Nrf2 (Fig. 8c, d). Transcriptome analyses of post-flight and ground control mouse tissues revealed that the expression of *Apoa4* and *Apoa5* was induced in the liver after spaceflight. Apolipoproteins contribute to lipid transport in plasma, and lipoproteins are abundant in lysophosphatidylcholines, phosphatidylcholines, and sphingomyelins^36^. We found that the expression of *Lcat* and *Agpat3* was also increased in the liver after spaceflight, and these genes were categorized into Class 1. LCAT catalyses the conversion of free cholesterol to cholesteryl ester and increases plasma cholesterol levels in mice^37^. In addition, ApoA4 enhances the activation of LCAT^38^. Therefore, the combined induction of LCAT and ApoA4 increases plasma cholesteryl ester levels. AGPAT3 exerts acyltransferase activity and catalyses the synthesis of phosphatidic acids^39^, a precursor for lipid biosynthesis. These results support the hypothesis that the induction of this set of genes contributes to the increased plasma lipid levels measured during spaceflight.

We also identified five Class 3 genes in the liver that may account for the specific metabolic changes in the plasma during spaceflight and the contributions of Nrf2 KO to these changes. Similarly, we surmise that the increase in eWAT in ground control Nrf2 KO mice may be attributable to the upregulation of three FA synthesis-related genes in Class 2, i.e.*, Scd1*, *Fasn* and *Elovl6*, in eWAT. These genes may also play roles in regulating the accumulation of triacylglycerols in GC-KO mouse eWAT. However, the expression of these genes is repressed in post-flight eWAT, suggesting that FA synthesis in eWAT is downregulated in post-flight mice.

Similarly, we have reported that the weights and lipid droplet sizes of eWAT were increased after the month-long flight in the MHU-3 project^24^. Therefore, the increase in eWAT volume in the post-flight mice is likely attributable to factors other than the increase in FA synthesis. An intriguing related observation is that the expression of *Abcg1* was substantially increased, but that of *Ces1f* in eWAT and triacylglycerol levels in plasma were significantly decreased after spaceflight. Although ABCG1 is best known as a cholesterol transporter, studies of *Abcg1* knockout mice revealed that ABCG1 also plays important roles in FA transport and lipid storage in adipocytes^40–42^. CES1F hydrolyses triacylglycerols^43,44^. Taken together, these observations suggest that in post-spaceflight mice, combined increases in FA uptake through increased ABCG1 expression and the suppression of triacylglycerol hydrolysis through the repression of CES1F expression led to increased weight and lipid droplet sizes in the eWAT.

We surmise that the following observations may also be pertinent to this discussion. After spaceflight, the expression of *Fasn* and *Scd1* was suppressed in eWAT, but the expression of *Acadm* and *Acadl* was induced in the liver. FASN and SCD1 catalyse the de novo synthesis and desaturation of FAs, respectively, while both ACADM and ACADL enhance the β-oxidation of FA in the liver. In addition, *Sfrp5* expression was increased in eWAT after spaceflight. *Sfrp5* encodes the SFRP5 protein, an adipokine that suppresses lipid accumulation^45^. A simple and straightforward interpretation of these observations would predict that the levels of FAs in eWAT and plasma would decrease due to suppressed FA synthesis and enhanced catabolism. However, plasma FA levels do not show a clear decrease during and after spaceflight. Thus, a 1-month stay in space might not be sufficient to induce the expected FA profile. Indeed, human studies have shown that long-term spaceflight decreases body weight and fat volume in astronauts^46^. The changes in the gene expression profile observed in this study should contribute to improving our understanding of the decreases in body weight that occur during space travel.

In the post-flight liver, the expression levels of *Fabp2*, *Cd36* and *Plin2* were markedly elevated. *Fabp2* knockout mice display increased plasma triacylglycerol levels^47^, as intestinal-type FA-binding protein I-FABP (product of *Fabp2*) decreases plasma triacylglycerol levels. CD36 is a multi-functional molecule^48^. CD36 overexpression increases FA uptake by the liver in mice^49^, and CD36 also functions as a thrombospondin-1 receptor^50^. The expression of *Thbs1*, the gene encoding thrombospondin-1, is also increased in eWAT after spaceflight. Thrombospondin-1 is an adipokine^51^ that exacerbates hepatic dysfunction^52^. Thus, the increased expression of CD36 in the liver and thrombospondin-1 in eWAT might synergistically affect biological functions during space travel. In addition, perilipin 2, encoded by *Plin2*, regulates lipid formation, and *Plin2*-null mice exhibit abnormalities in plasma triacylglycerol levels^53^. Therefore, the changes in *Plin2* expression may contribute to the decrease in plasma triacylglycerol levels observed after spaceflight.

In this study, we also found that the expression of a number of endocrine-related genes were affected by spaceflight. For instance, the expression of *Dio2*, which encodes type II iodothyronine deiodinase (D2), was decreased, while the expression of *Cort* (encoding cortistatin) was increased in the cerebrum after spaceflight. The expression of the latter gene is also regulated by Nrf2. The changes in D2 and cortistatin expression seem pertinent to the changes in lipid metabolism observed during spaceflight, as D2 contributes to the conversion of the prohormone thyroxine to the active form triiodothyronine, which is known to regulate tissue phospholipid levels^54^. Similarly, cortistatin is a somatostatin-related cyclic neuropeptide that regulates insulin secretion^55^. In addition, the expression levels of *Epor, Mc2r* and *Oxtr*, which encode receptors of erythropoietin, adrenocorticotropic hormone (ACTH) and oxytocin, respectively, were increased in eWAT after spaceflight. We speculate that these changes may also be related to the observed metabolic changes, especially since erythropoietin signalling is reported to regulate lipid droplet size^46^ and erythropoietin receptor contributes to preadipocyte differentiation^56^. Similarly, although the roles of ACTH and oxytocin receptors in signalling within eWAT have not been fully clarified, ACTH is known to regulate metabolism in brown adipose tissue^57^, and oxytocin receptor signalling exerts insulin-like activity in adipocytes^58^. However, the effects of spaceflight on endocrine systems and their contributions to the regulation of lipid metabolism remain to be clarified.

In addition to metabolism- and endocrine system-related genes, spaceflight induces the expression of the antioxidant and detoxification enzyme gene *Gstm1* in eWAT in an Nrf2-dependent manner. Although GST mu1 is known to play important roles in detoxification^59^, it also increases the expression of uncoupling protein 1 and contributes to metabolic regulation in mice^60^. Therefore, upregulation of *Gstm1* gene expression might contribute to both detoxification and metabolic regulation.

Based on our findings, Nrf2 is involved in metabolic responses during and after spaceflight. Nrf2-inducing chemicals are used for the treatment of multiple sclerosis^61^ or are in clinical trials for diabetic kidney disease^62^. An Nrf2-inducing chemical protects metabolism-related organs and tissues and contributes to maintaining metabolic homeostasis^63,64^. Although we have not experimentally examined whether the activation of Nrf2 signalling actually modulates the metabolic response in spaceflight, the available evidence suggests that Nrf2 induction is useful for adaptation to the space environment.

In conclusion, these findings show that space stresses induce metabolic changes in the plasma and eWAT. The altered expression of metabolism-related genes suggests that a metabolic response in plasma occurs in space travel as a result of gene regulation in the eWAT and liver. In addition, the lipid metabolic changes in eWAT after spaceflight are strongly correlated with eWAT lipid droplet size, indicating that these metabolic changes indeed contribute to the health problems observed during space travel. This study also provides important evidence for the contributions of Nrf2 to the metabolic response to space stresses, and Nrf2 may be a new target to maintain metabolic homeostasis during space travel for astronauts, tourists and settlers.

## Methods

### MHU-3 project

The design of the MHU-3 project was reported previously^24^. A transportation cage unit (TCU) was used to transport mice aboard the SpaceX Dragon capsule during the launch and return phases. A 12 h:12 h day:night cycle was established in the habitat cage unit (HCU) aboard the ISS. Male Nrf2 KO (Nfe212tm1Ymk) and WT mice on a C57BL/6J background were bred at Charles River Laboratories Japan. All animal experiments were approved by the Institutional Animal Care and Use Committees of JAXA (protocol numbers 017-001 and 017-014), NASA (protocol number FLT-17-112), and Explora BioLabs (EB15-010C).

### FL and GC experiments

MHU-3 FL (flight) and GC (ground control) experiments were performed as previously reported^24^. Three weeks prior to launch, 8-week-old mice were delivered from Charles River Laboratories Japan. Mice were loaded into the TCU and transported to the ISS by SpX14 and then transferred to the ISS HCU. For return to Earth, mice were transferred to the TCU aboard SpX14 and splashed down in the Pacific Ocean. The mice were retrieved and transported to Explora BioLabs in San Diego. The GC experiment that paralleled the space experiment was conducted at JAXA Tsukuba in Japan. Six WT and six Nrf2 KO mice were individually housed in the same manner as for the FL experiment. Blood samples were collected from the tail at three time points, immediately centrifuged at 1580 × *g* for 10 min at 4 °C, frozen and stored at the respective sites before measurement. Blood samples were also collected from IVCs after spaceflight. The samples were centrifuged immediately at 1200 × *g* for 15 min at 4 °C, and the supernatants were aliquoted, frozen and stored.

### RNA-seq analysis

RNA-seq analyses were conducted as previously reported^24^. Total RNA was isolated from the liver, eWAT and cerebrum. RNA samples (1.0 μg) were subjected to isolation of poly(A)-tailed RNA and library construction using the Sureselect Strand-Specific RNA Sample Prep Kit (Agilent Technologies). Raw sequence reads were mapped to the mouse mm10 genome using STAR (version 2.6.1). TPM values were obtained to measure gene expression using RSEM (version 1.3.1). The TPM was normalized using Subio Platform software.

### Metabolome analyses

Metabolome analyses of tail plasma samples were performed using the MxP Quant 500 Kit (Biocrates Life Sciences) according to the manufacturer’s instructions^65^. Tail plasma samples (5 µL) were used for the UHPLC-MS/MS analyses (Xevo TQ-S system, Waters). The eWAT samples used for the lipidomics analysis were stored at −80 °C until LC-MS analysis. eWAT samples of 10–50 mg were homogenized in the optimal volume of methanol (10 μL per mg of tissue) and prepared using a previously reported method^66^; the eWAT extracts were then subjected to UHPLC-FTMS (QExactive, Thermo Fisher Scientific). The UHPLC conditions were modified from a previously reported method^67^. Separation was performed using a metal-free C18 column (L-column2 ODS, 2.0 mm i.d. × 100 mm, 2-µm particle size; CERI). The mobile phase consisted of 60/40/1 (v/v/v%) acetonitrile/water/ammonium formate (1 mol/L) containing 0.1% formic acid (A) and 10/90 (v/v%) acetonitrile/isopropanol containing 0.1% formic acid (B). Metabolites were separated with a gradient; the initial condition was 30% B at a rate of 0.2 mL/min, followed by a linear gradient to 100% B from 2.0 to 20.0 min and 100% B for 10.0 min. Then, the mobile phase was returned to the initial condition and maintained for 5.0 min until the end of the run. The total run time was 35.0 min. The temperature of the column compartment was 45 °C. The FTMS system was equipped with a heated ESI-II source. The voltages used in positive and negative ion modes were 3.5 and 2.5 kV, respectively. The heated capillary temperature was 275 °C, the sheath gas pressure was 45 psi, the auxiliary gas setting was 10 psi, and the heated vaporizer temperature was 300 °C. Both the sheath gas and auxiliary gas were nitrogen. The collision gas was argon at a pressure of 1.5 mTorr. The FTMS scan type was full MS/data-dependent (dd)-MS^2^. The parameters of the full mass scan were as follows: resolution 70,000, autogain control target under 1 × 10^6^, maximum isolation time 100 ms, and *m/z* range 350–1050. The parameters of the dd-MS^2^ scan were as follows: resolution 17,500, autogain control target under 1 × 10^5^, maximum isolation time 50 ms, loop count 5, number of top peaks 5, isolation *m/z* window 1.5, normalized collision energy 30, underfill ratio 5.00%, and intensity threshold <1 × 10^5^. The UHPLC-FTMS system was controlled by Xcalibur 4.2.28.14 software (Thermo Fisher Scientific), and data were collected with the associated software. The chemical standards of lipid species were obtained from Avanti Polar Lipids and analysed using UHPLC-FTMS to confirm the adduct ion for each group of lipid species, to obtain the fragment ion mass spectra for identification of the fatty acids of lipid species, and to provide the retention time information needed to discriminate species with different numbers of carbons or double bonds within the same group of lipids.

IVC plasma metabolites were evaluated using NMR spectroscopy^24^. For this purpose, plasma metabolites were extracted from 50 µL of plasma using a standard methanol extraction method. NMR experiments were performed at 298 K on a Bruker Avance 600-MHz spectrometer equipped with a CryoProbe and a SampleJet sample changer.

### MALDI-MSI

Mouse brain samples were frozen in liquid nitrogen and stored at −80 °C. MALDI-MSI analyses were conducted as previously reported^34,68^. Frozen brain tissues were dissected for cryosectioning at 8-μm thickness using a CM 3050S cryostat (Leica Microsystems). Sections were thaw-mounted on indium-tin oxide slides (100 ohm/sq; Matsunami). A α-cyano-4-hydroxycinnamic acid (CHCA, Sigma-Aldrich) matrix with a thickness of 0.7 μm was applied to the specimens using an iMLayer (Shimadzu). MALDI-MSI analysis was performed with an iMScope (Shimadzu), and MS spectra were acquired by pulsing the sample with 100 laser shots per data point in positive-ion mode. The diameter of laser irradiation was 25 μm, and the spatial interval between each data point was 70 μm. The data were processed with Imaging MS solution v1.30 analysis software (Shimadzu).

### Statistics and reproducibility

Data are presented as the means ± standard deviations (SDs). Statistical analyses were performed using Student’s *t*-test, and the *P* values of Student’s *t*-test were adjusted using the Holm method^69^. For multiple comparisons, analysis of variance (ANOVA) followed by Tukey’s post hoc test were performed. Differences were considered statistically significant at an adjusted *P* < 0.05. Multivariate analyses of eWAT metabolite levels *vs*. lipid droplet size were performed using JMP Pro software (Ver. 15.0.0).

### Reporting summary

Further information on research design is available in the Nature Research Reporting Summary linked to this article.

## Supplementary information

Supplementary Information

Description of Additional Supplementary Files

Supplementary Data 1

Supplementary Data 2

Supplementary Data 3

Supplementary Data 4

Supplementary Data 5

Supplementary Data 6

Supplementary Data 7

Supplementary Data 8

Reporting Summary

## Data Availability

The metabolome and RNA-seq data in this study have been deposited in the ibSLS (Integrated Biobank for Space Life Science, https://ibsls.megabank.tohoku.ac.jp/)^5^. The RNA-seq data in this study also have been deposited in Gene Expression Omnibus of NCBI (accession number GSE152382). All relevant data are available from the corresponding authors upon reasonable request. Source data for graphs and charts presented in the main figures are provided in Supplementary Data 8.
