# Supplementary material for: Nrf2 plays a critical role in the metabolic response during and after spaceflight
Source: Commun Biol. 2021 Dec 9;4:1381. doi: 10.1038/s42003-021-02904-6 (PMC8660801; doi:10.1038/s42003-021-02904-6)
Supplement: Supplementary file 1 — Supplementary Information [file 42003_2021_2904_MOESM1_ESM.pdf]

Supplementary Figure 1

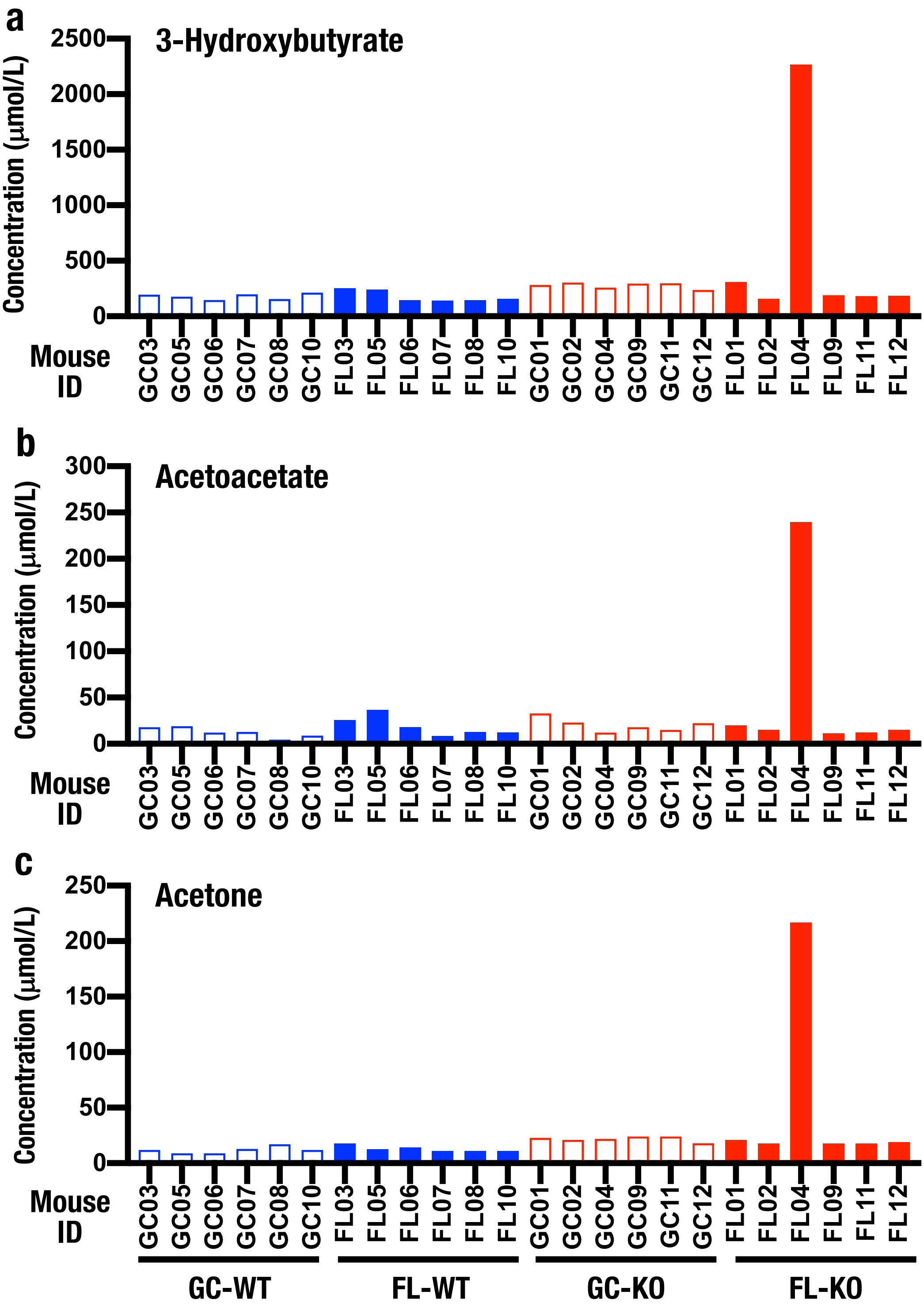

Supplementary Fig. 1 Plasma ketone body levels. a-c, IVC plasma concentrations of ketone bodies, including 3-hydroxybutyrate (**a**), acetoacetate (**b**) and acetone (**c**), determined by NMR at R+2. The results are presented as the plasma concentrations in individual mice.

Supplementary Figure 2

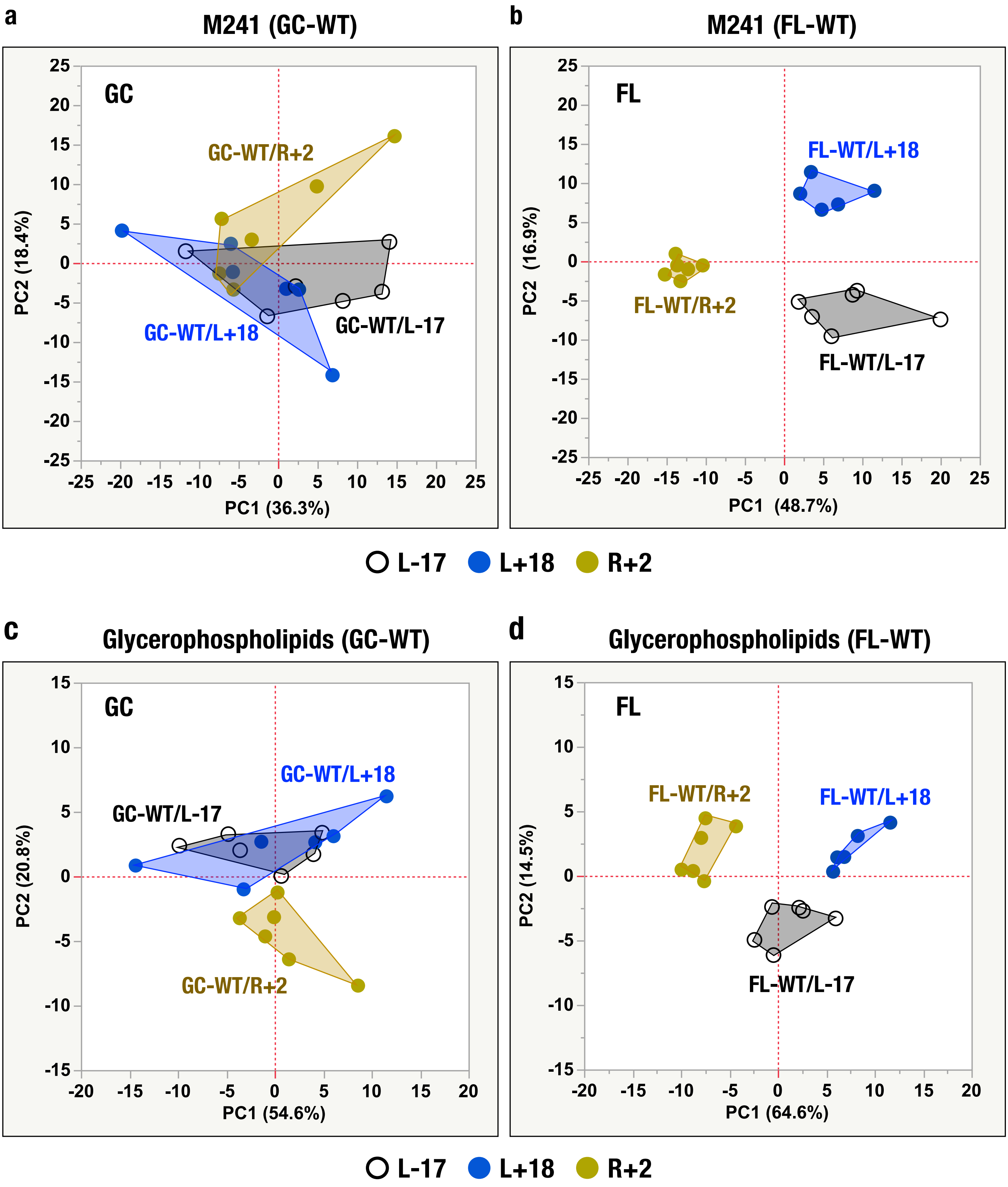

Supplementary Fig. 2 PCA of plasma metabolome. a-d, Plots of PCA for M241 dataset (**a** for GC-WT and **b** for FL-WT) and 72 glycerophospholipids (**c** for GC-WT and **d** for FL-WT). GC-WT (n=6 each) and FL-WT (n=6 for L-17 and R+2; n=5 for L+18).

Supplementary Figure 3

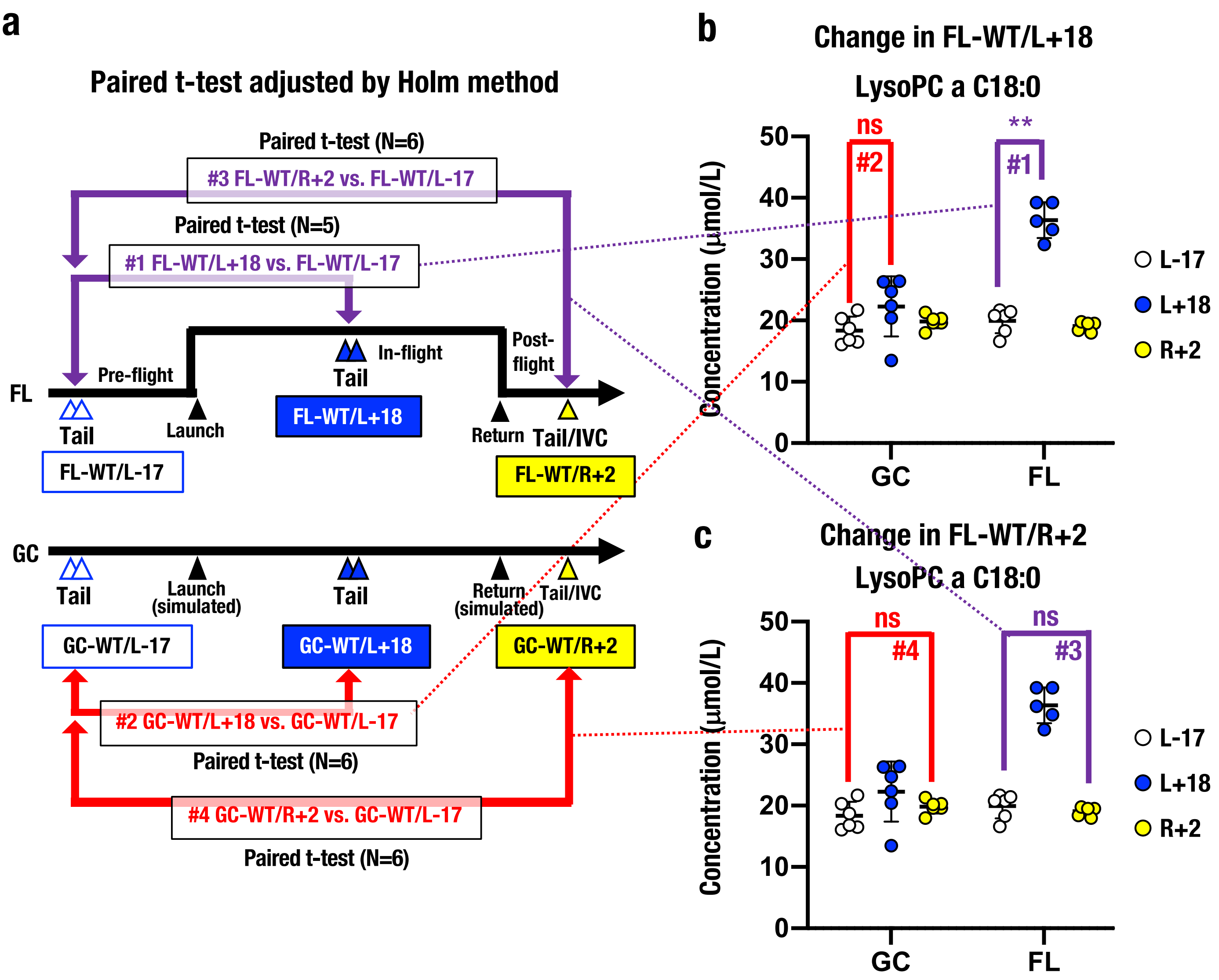

d

Group	A	B	C	D	E	F	G	H	Total
Amino acids	0	0	0	0	1	0	0	19	20
Amino acid-Related	0	0	0	0	3	2	2	16	23
Glycerophospholipids	15	15	2	0	0	0	12	28	72
Sphingolipids	1	1	4	1	0	0	0	4	11
Triacylglycerols	0	0	0	0	0	2	34	33	69
Other metabolites	1	1	0	1	0	1	10	32	46
Total	17	17	6	2	4	5	58	132	241
Change in FL-WT/L+18	Inc	Inc	Inc	NS	Dec	Dec	NS	NS	
Change in FL-WT/R+2	NS	Dec	Inc	Inc	NS	Dec	Dec	NS	

Supplementary Figure 3

Supplementary Fig. 3 Metabolic changes in WT mice of dataset M241. a, Statistical analyses to identify altered metabolites in FL-WT mice. Paired t-tests were performed between timepoints (L+18 *vs.* L-17, #1 for FL-WT and #2 for GC-WT; R+2 *vs.* L-17, #3 for FL-WT and #4 for GC-WT) and *P* values were adjusted by Holm method. **b**-**d**, The metabolites altered in FL-WT mice at L+18 and R+2 were determined by combinations of statistical analyses (#1 and #2 for L+18; #3 and #4 for R+2) as adjusted $P<0.05$. Plasma levels of LysoPC a C18:0, a representative metabolite altered at L+18, are shown in **b** (for L+18) and **c** (for R+2). Results are presented as the mean ± SD of plasma levels (μmol/L), and statistical analyses were performed using paired t-tests and *P* value was adjusted by Holm method. **Adjusted $P<0.01$. *ns*, not significant. Numbers of increased (Inc) or decreased (Dec) metabolites during and after spaceflight (**d**). Metabolite classes are described in Supplementary Data 1 and 2.

Supplementary Figure 4

a

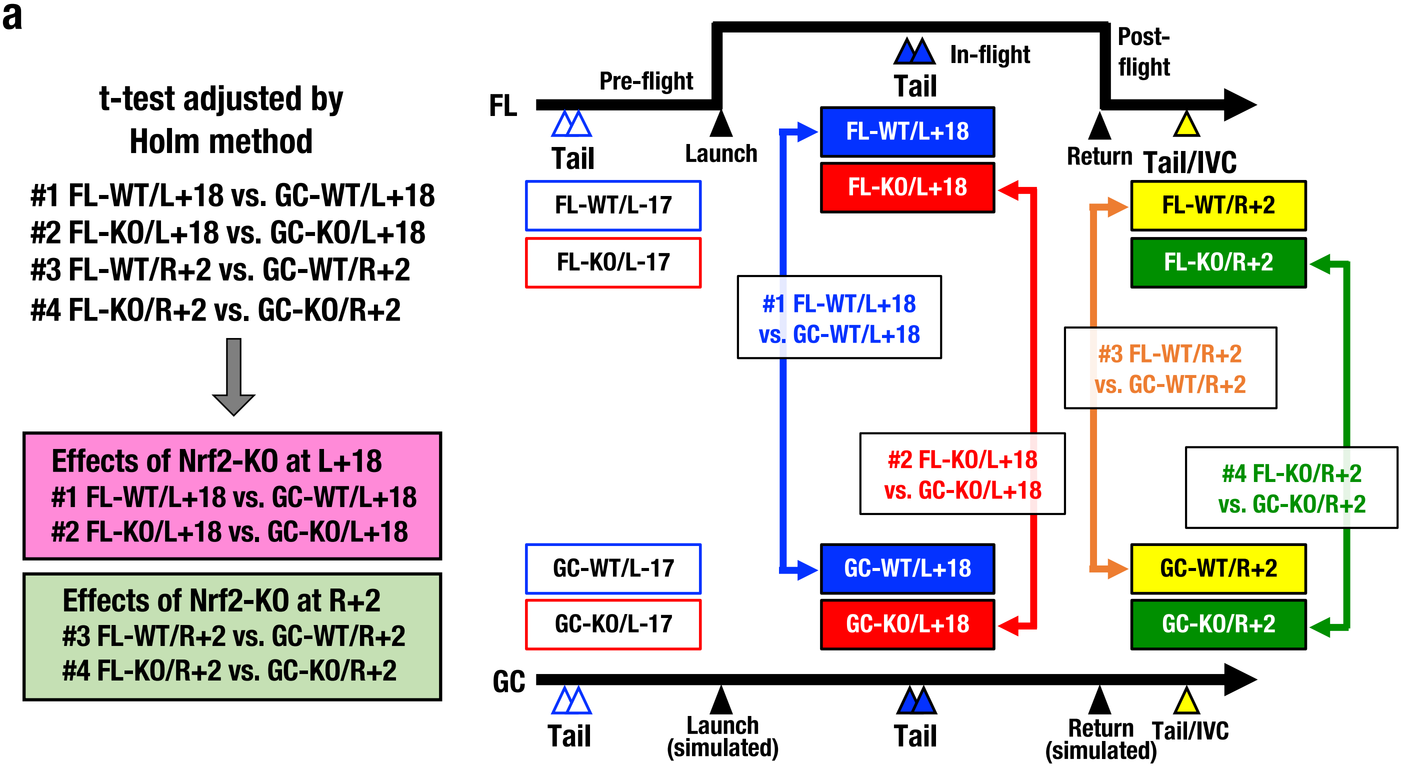

b Numbers of altered metabolites in FL-WT and FL-KO at L+18

Group	A	B	C	D	E	F	G	H	Total
Amino acids	1	0	1	0	1	4	0	13	20
Amino acid-Related	1	0	2	0	2	2	0	16	23
Glycerophospholipids	16	2	7	0	0	0	7	40	72
Sphingolipids	5	0	1	0	0	0	0	5	11
Triacylglycerols	7	0	0	0	0	1	7	54	69
Other metabolites	12	0	5	0	0	0	0	29	46
Total	42	2	16	0	3	7	14	157	241
Change in FL-WT/L+18	Inc	Inc	Inc	NS	Dec	Dec	NS	NS	
Change in FL-KO/L+18	NS	Dec	Inc	Inc	NS	Dec	Dec	NS	

c Numbers of altered metabolites in FL-WT and FL-KO at R+2

Group	I	J	K	L	M	N	O	Total
Amino acids	0	1	1	4	0	0	14	20
Amino acid-Related	0	1	1	4	1	0	16	23
Glycerophospholipids	3	1	0	22	19	3	24	72
Sphingolipids	2	2	0	0	1	0	6	11
Triacylglycerols	0	0	0	15	0	0	54	69
Other metabolites	5	2	0	4	3	1	31	46
Total	10	7	2	49	24	4	145	241
Change in FL-WT/R+2	Inc	Inc	NS	Dec	Dec	NS	NS	
Change in FL-KO/R+2	NS	Inc	Inc	NS	Dec	Dec	NS	

Supplementary Figure 4

Supplementary Fig. 4 Metabolic changes in dataset M241 in Nrf2 KO mice. **a**, Statistical analyses to identify altered metabolites in FL-KO mice. t-Test was performed for comparing FL *vs*. GC at L+18 (#1 for WT and #2 for KO) and R+2 (#3 for WT and #4 for KO) and the *P* values were adjusted by Holm method. The metabolites altered in FL-KO mice at each timepoint were then determined by a combination of #1 and #2 for L+18 (magenta box) and a combination of #3 and #4 for R+2 (green box) as adjusted $P<0.05$. **b**,**c**, Numbers of increased (Inc) or decreased (Dec) metabolites in FL-WT and FL-KO at L+18 (**b**) and R+2 (**c**). Metabolite classes are described in Supplementary Data 1 and 3.

Supplementary Figure 5

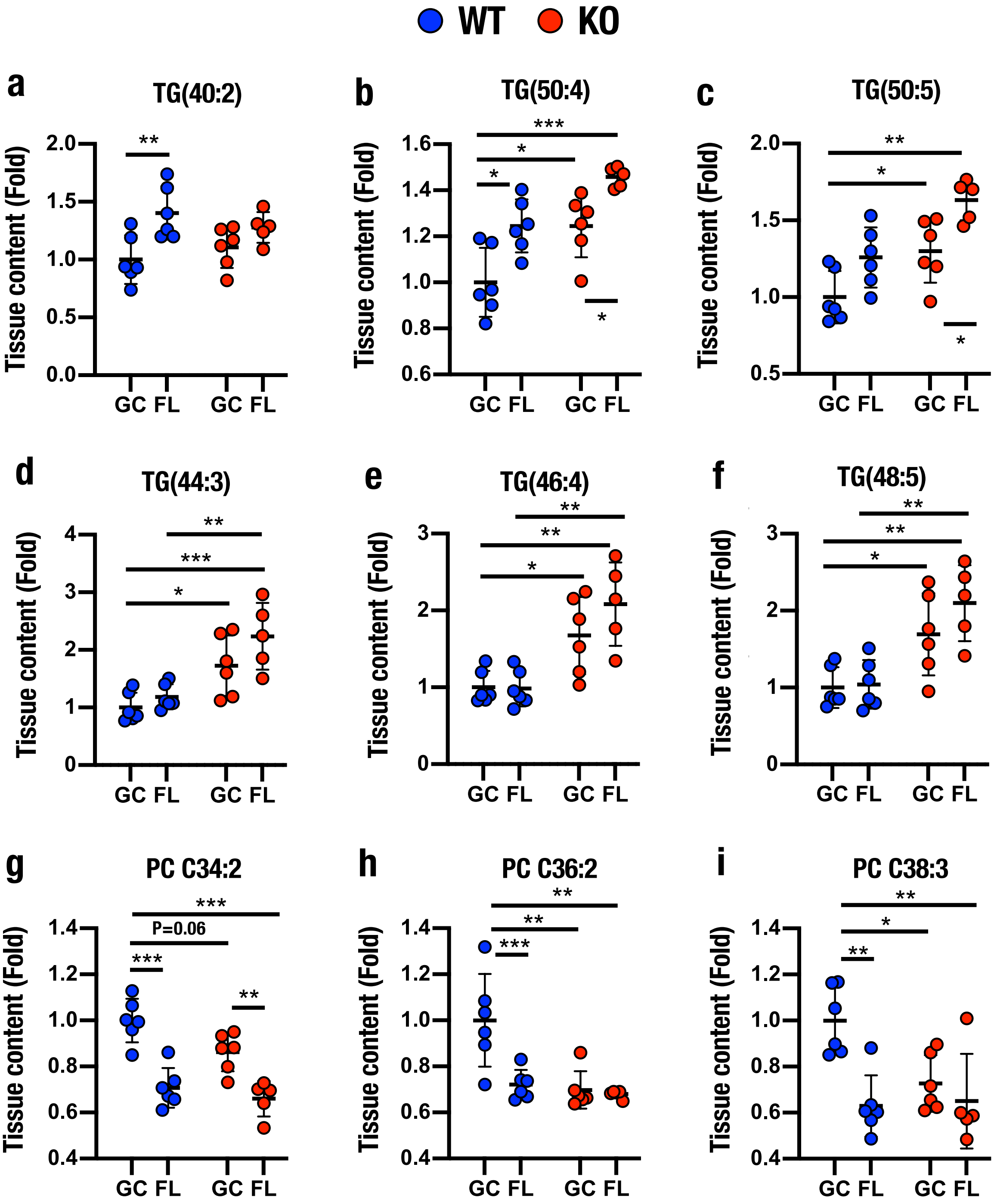

Supplementary Fig. 5 Lipid levels in eWAT. a-i, Lipidomics analyses of eWAT. Concentrations of triacylglycerols, including TG(40:2) (**a**), TG(50:4) (**b**) TG(50:5) (**c**), TG(44:3) (**d**), TG(46:4) (**e**) and TG(48:5) (**f**), and phosphatidylcholines, including PC C34:2 (**g**), PC C36:2 (**h**), PC C38:3 (**i**), in the eWAT. The results are presented as the mean ± SD, and the mean in GC-WT was set as one. Statistical analyses were performed using ANOVA followed by Tukey post hoc test. **P*<0.05, ***P*<0.01 and ****P*<0.001.

Supplementary Figure 6

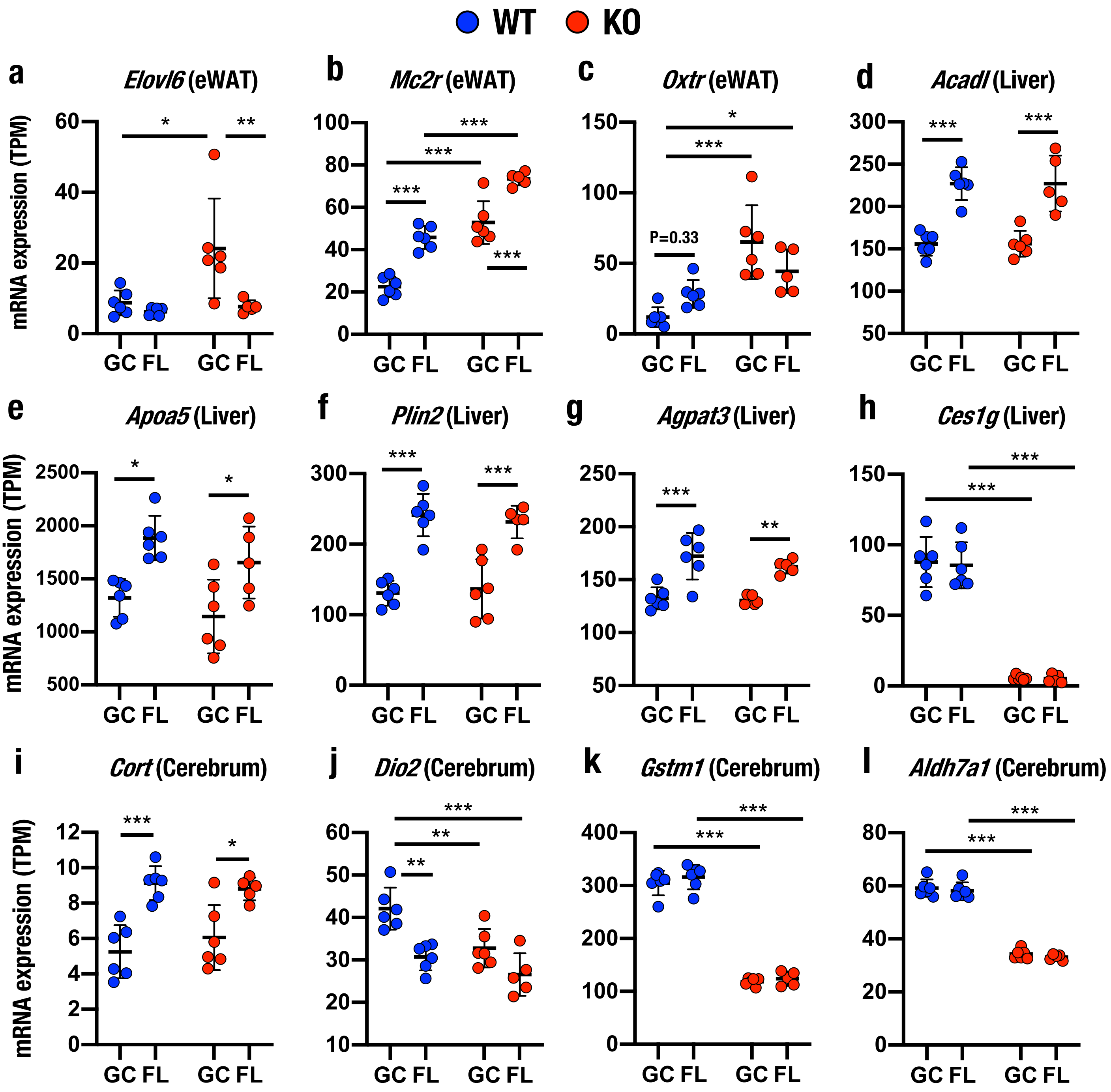

Supplementary Fig. 6 Altered gene expression in the eWAT, cerebrum and liver during spaceflight and Nrf2 KO. a-h, Expression levels of representative differentially expressed genes in the eWAT (**a-c**), liver (**d-h**) and cerebrum (**i-l**) as determined by RNA-seq. Expression levels of *Elovl6* (**a**), *Mc2r* (**b**), *Oxtr* (**c**), *Acadl* (**d**), *Apoa5* (**e**), *Plin2* (**f**), *Agpat3* (**g**), *Ces1g* (**h**), *Cort* (**i**), *Dio2* (**j**), *Gstm1* (**k**) and *Aldh7a1* (**l**). The results are presented as the mean ± SD of the gene expression levels (TPM). Statistical analyses were performed using ANOVA followed by Tukey post hoc test. **P*<0.05, ***P*<0.01 and ****P*<0.001.

Supplementary Figure 7

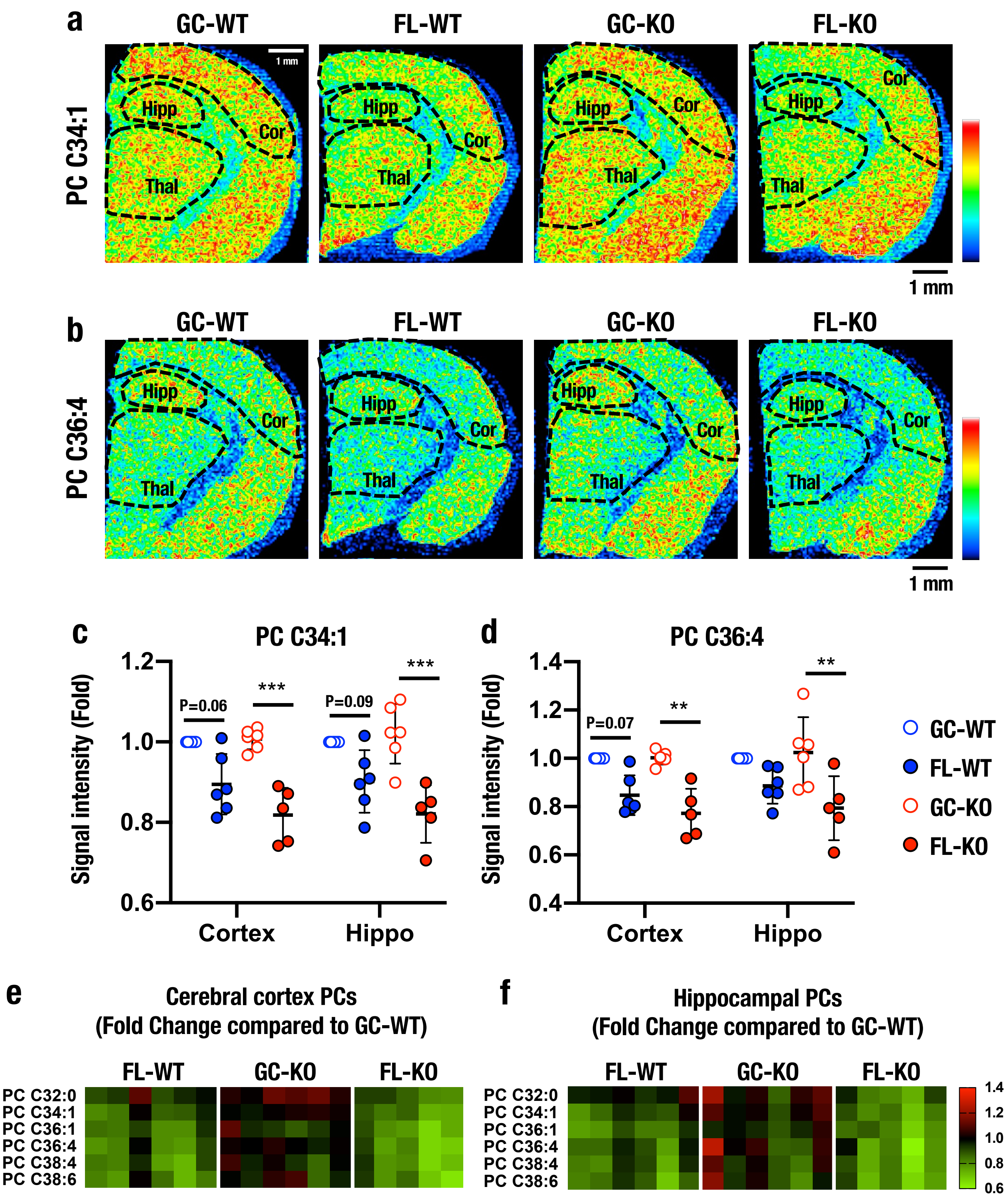

Supplementary Figure 7

Supplementary Fig. 7 Phosphatidylcholine levels in the brain after spaceflight. a,**b**, MALDI-MSI analysis of phosphatidylcholines in the brain. MS images of PC 34:1 (**a**) and PC 36:4 (**b**) signals in brain sections of mice at positions 8 mm posterior to bregma. The scale bars represent 1 mm. *Cor*, cerebral cortex. *Hipp*, hippocampus. *Thal*, thalamus. **c,d** Quantification of MALDI-MSI analyses of PC C34:1 (**c**) and C36:4 (**d**) in the cerebral cortex and hippocampus. MALDI-MSI analyses were performed with one GC-WT (n=6), FL-WT (n=6), GC-KO (n=6) and FL-KO (n=5) mouse per batch. MSI signal intensities in the cortex and hippocampus were quantified, and the signal of GC-WT mice was set as 1 in each batch. Then, the relative signal intensities were pooled in 6 independent experiments. The results are presented as the mean ± SD. Statistical analyses were performed using ANOVA followed by Tukey post hoc test. ***P*<0.01 and ****P*<0.001. **e,f** Heatmap data analyses of MALDI-MSI analysis of 6 phosphatidylcholines in the cerebral cortex (**e**) and hippocampus (**f**), including PC C32:0, C34:1, C36:1, C36:4, C38:4 and C38:6. MSI signal intensities were quantified, and the signal from GC-WT mice was set as 1 in each batch. The results are presented as the fold change compared to GC-WT. FL-WT (n=6), GC-KO (n=6), FL-KO (n=5).
