# Supplementary material for: Nrf2 plays a critical role in the metabolic response during and after spaceflight
Source: Commun Biol. 2021 Dec 9;4:1381. doi: 10.1038/s42003-021-02904-6 (PMC8660801; doi:10.1038/s42003-021-02904-6)
Supplement: Supplementary file 2 — Description of Additional Supplementary Files [file 42003_2021_2904_MOESM2_ESM.pdf]

Description of Additional Supplementary Files

File name: Supplementary Data 1.
Description: Classifications and metabolite numbers for datasets M624, M241 and M104.

File name: Supplementary Data 2.
Description: Statistical results of datasets M241 and M104. Changes in FL-WT/L+18 and FL-WT/R+2.

File name: Supplementary Data3.
Description: Statistical results of datasets M241 and M104. Changes in FL-WT/L+18, FL-WT/R+2, FL-KO/L+18 and FL-KO/R+2.

File name: Supplementary Data 4.
Description: Statistical results of RNA-seq analyses in eWAT.

File name: Supplementary Data 5.
Description: Statistical results of RNA-seq analyses in liver.

File name: Supplementary Data 6.
Description: Statistical results of RNA-seq analyses in Cerebrum.

File name: Supplementary Data 7.
Description: Representative genes altered in RNA-seq analyses.

File name: Supplementary Data 8.
Description: Source data for graphs and charts presented in the main figures.
